# miR-889-3p targeting BMPR2 promotes the development of retinoblastoma via JNK/MAPK/ERK signaling

**DOI:** 10.1038/s41598-023-49994-2

**Published:** 2024-03-27

**Authors:** Yuan Gao, Pei Du

**Affiliations:** Department of Ophthalmology, Wuhan No. 1 Hospital, No. 215, Zhongshan Avenue, Qiaokou District, Wuhan, 430022 Hubei China

**Keywords:** Cancer, Cell biology

## Abstract

MicroRNAs (miRNAs) are vital regulators of tumor pathogenesis, including that of retinoblastoma (Rb). This study investigated the functions and mechanisms of action of miR-889-3p in Rb. BMPR2 and miR-889-3p levels were assessed by quantitative reverse transcription PCR (qRT-PCR) or western blotting. Through several cell function tests, the effects of miR-889-3p and BMPR2 on cell proliferation, migration, and JNK/MAPK/ERK signaling were evaluated. The interaction between miR-889-3p and BMPR2 was investigated using a luciferase reporter assay. In vivo tumor development was investigated using a xenograft test. The association between miR-889-3p and BMPR2 expression was identified using Pearson’s correlation analysis. miR-889-3p was increased in Rb cells, and miR-889-3p knockdown inhibited Rb cell proliferation, migration, and phosphorylation of c-Jun N-terminal kinase (JNK), p38 mitogen-activated protein kinase (MAPK), and ERK1/2 in vitro, as well as tumor growth in vivo. Further, they were inversely associated in Rb tissues and miR-889-3p may directly attached to the 3′-UTR of BMPR2 mRNA. Finally, the inhibition of BMPR2 inverted the negative effects of the miR-889-3p inhibitor on migration, proliferation, and activation of JNK, p38 MAPK, and ERK1/2 in Rb cells. Our results indicate that miR-889-3p, which targets BMPR2 and promotes Rb growth by controlling the JNK/MAPK/ERK pathway, is an oncogene in Rb. These results suggested that the miR-889-3p/BMPR2 axis may be a new therapeutic target for Rb.

## Introduction

Retinoblastoma (Rb) is the most common intraocular malignant tumor in infants and young children^1^. The primary treatment options for patients with R are photocoagulation, laser therapy, enucleation, chemotherapy, and targeted therapy^2^. Due to the rapid progression of the disease, the prognosis remains poor despite substantial advancements in Rb diagnosis and therapy over the past ten years^3^. Moreover, most patients are diagnosed with Rb at advanced stages; therefore, the optimal treatment period has not passed, resulting in poor treatment outcomes, particularly in developing countries^4^. Accordingly, discovering molecular diagnostic and therapeutic methods for patients with Rb is necessary, which requires an understanding of the molecular mechanisms of Rb.

MicroRNAs (miRNAs) are a class of endogenous noncoding small RNA molecules that influence the expression of downstream target genes in a highly conserved manner^5^. miRNAs can mediate mRNA degradation and/or suppression of transcription by targeting the 3′-UTR regions of their target genes; therefore, they participate in many physiological processes and pathologies, such as carcinogenesis and the progression of cancer^6–8^. Numerous studies conducted in recent years have discovered that numerous miRNAs are inappropriately expressed in Rb, which can either promote or hinder Rb development^9,10^. For instance, miR-142-5p is downregulated in Rb cells and attenuates Rb cell malignancy^11^. Therefore, identifying abnormally expressed miRNAs and their target genes in Rb will open new avenues for research on the molecular causes of this disease and potential therapies. MiR-889-3p has been shown to act as a tumor suppressor in cervical cancer^12,13^ and hepatocellular carcinoma^14^ or as an oncogene in malignant peripheral nerve sheath tumors and osteosarcoma^15,16^. However, the role of miR-889-3p in Rb remains unclear.

Bone morphogenetic protein receptor 2 (BMPR2) is related to the apoptosis, proliferation, and metastasis of tumor cells^17–19^. In liver cancer, *OIP5* knockdown upregulates BMPR2 expression to inhibit the progression of liver cancer, suggesting an anticancer role for BMPR2 in liver cancer^20^. However, BMPR2 enhances ovarian cancer progression, indicating its oncogenic role of BMPR2 in ovarian cancer^21^. These studies revealed the different roles of BMPR2 in different cancers. In addition, excessive BMPR2 expression can prevent osteoarthritis by activating the MAPK/JNK/ERK signaling pathway^22^. It remains unclear how much BMPR2 regulates the JNK/MAPK/ERK signaling pathway activity in Rb.

MiR-889-3p was found to be an oncogene in Rb. In addition, we verified that miR-889-3p specifically targets BMPR2 and suppresses its expression, which increases JNK/MAPK/ERK signaling. These findings provide novel insights into Rb and may help identify potential diagnostic and therapeutic targets.

## Materials and methods

### Tissue samples

We collected 37 Rb samples and matched normal tissues from our hospital. None of the patients had received radiation or chemotherapy before surgery. This study was approved by the ethics committee of Wuhan No. 1 Hospital and informed consent has been obtained from the guardians of all patients. Clinical tissue samples were handled according to the ethical standards outlined in the Declaration of Helsinki.

### Cell culture

RPMI-1640 medium (Catalog number: 11875168) obtained from Gibco (Grand Island, NY, USA), supplemented with 10% FBS (Catalog number: 26140, Gibco), was used to sustain the Rb cell lines (SO-RB50, Y79, and HXO-RB44) and the normal human retinal epithelial cell line ARPE-19. ARPE-19 and Y79 cells were purchased from the American Type Culture Collection (ATCC, Rockville, MD, USA), SO-RB50 was developed at the Zhongshan Ophthalmic Center (ZOC), and HXO-RB44 was obtained from the Cell Culture Center, Xiangya Medical College of Central South University. The cells were incubated at 37 °C in a humidified environment with 5% CO_2_.

### Cell transfection

The miR-889-3p mimics/inhibitor, negative control (NC) mimics/inhibitors, si-BMPR2, and si-NC were developed and acquired from GenePharma Co., Ltd. (Shanghai, China). These oligo fragments were introduced into cells by transfection with Lipofectamine 2000 (catalog number: 11668027, Invitrogen, Carlsbad, CA, USA) in accordance with the manufacturer’s instructions. Cells in the si-BMPR2 + miR-889-3p inhibitor group were co-transfected with si-BMPR2 and the miR-889-3p inhibitor using Lipofectamine 2000. Cells were collected for subsequent experiments 24 h after transfection.

### Quantitative RT-PCR (qRT-PCR)

Following the manufacturer’s instructions, total RNA was extracted from tissues and cells using TRIzol reagent (catalog number: 15596-026, Invitrogen). cDNA was reverse-transcribed using the PrimeScript RT Reagent kit (Catalog number: RR037A) from Takara (Dalian, China). For qRT-PCR, a SYBR Green PCR kit (Catalog number: RR820A; Takara) was used. Each gene of interest was amplified using a specific set of primers. Data were calculated using the relative quantitation (2^−ΔΔCt^) technique, and each experiment was performed at least thrice. Real-time PCR primer sequences are listed in Table 1.

**Table 1:** Real-time PCR primer sequences.

Gene name	Sequence
miR-889-3p	Forward 5′–GCCGAGTTAATATCGGACAA–3′
	Reverse 5′–CAGTGCGTGTCGTGGAGT–3′
BMPR2	Forward 5′–CACCTCCTGACACAACACCACTC–3′
	Reverse 5′–TGCTGCTGCCTCCATCATGTTC–3′
GAPDH	Forward 5′–CCTTCATTGACCTCAACTAC–3′
	Reverse 5′–CTCCTGGAAGATGGTGATGG–3′
U6	Forward 5′–GCTTCGGCAGCACATATACTAAAAT–3′
	Reverse 5′–CGCTTCACGAATTTGCGTGTCAT–3′

### Cell counting Kit-8 assay (CCK8)

Cells were placed in 96-well plates at a density of 3 × 10^3^ cells per well in 100 µL of RPMI 1640 medium supplemented with 10% FBS to transfect the oligo fragments into the cells. Then, CCK-8 reagent (10 µL, Catalog number: C0037, Beyotime, Shanghai, China) was supplemented to each well and the 96-well plate was placed in 5% CO_2_ at 37 °C for 1 h. Microplate Readers (Molecular Devices, Sunnyvale, CA, USA) were used to measure the OD_450_ values. Each experiment was performed at least thrice.

### Scratch test

Scratching experiments were performed to quantify the cell migration. Trypsinized cells were seeded onto 6-well plates with three parallel wells per group and cultivated until 90% confluence. The cells were then maintained in the FBS-free media while having vertical scratches made with a 100 µL tip. Under an inverted microscope, the cells were examined at 0 h and 24 h, and the migration rate was calculated using the formula (0 h scratch width–24 h scratch width)/0 h scratch width × 100%.

### Western blotting

RIPA buffer was used to lyse total proteins from the cells (catalog number: P0013B, Beyotime, Shanghai, China). A BCA Protein Quantification Kit (catalog number: KTD3001, Abbkine, Atlanta, Georgia, USA) was used to measure protein concentrations. PVDF membranes (Millipore, Billerica, MA, USA) were used after the proteins were transferred after being separated by 10% sodium dodecyl sulfate–polyacrylamide gel electrophoresis. Primary antibodies were used to hatch the membranes after they had been inhibited with 5 percent skim milk for JNK (Catalog number: 9252, 1:1000; Cell Signaling Technology, Danvers, MA, USA), phosphorylated JNK (p-JNK, Catalog number: 8690, 1:1000; Cell Signaling Technology), p38 MAPK (Catalog number: 4668, 1:1000; Cell Signaling Technology), phosphorylated p38 MAPK (Catalog number: 9216, 1:1000; p-p38 MAPK, Cell Signaling Technology), ERK1/2 (Catalog number: 4695, 1:1000; Cell Signaling Technology), phosphorylated ERK1/2 (p-ERK1/2, Catalog number: 8544, 1:1000; Cell Signaling Technology), BMPR2 (Catalog number: ab96826, 1:1000; Abcam, USA), and GAPDH (Catalog number:5174, 1:1000; Cell Signaling Technology) while temperature applied were 4 °C for overnight. Following an hour of exposure to the appropriate secondary antibodies, the membranes were visualized using ChemiDoc digital imaging equipment (Bio-Rad). ImageJ software was used to quantify the western blot analysis results.

### RNA pull-down assay

An RNA pull-down kit (Catalog number: KT103-01, Guangzhou Saicheng Biotechnology Co., LTD, Guangzhou, China) was used to perform the assay. Biotinylated miR-889-3p (Bio-miR-889-3p) and its negative control (bio-NC) were purchased from GenePharma Co., Ltd. (Shanghai, China) and transfected into HXO-RB44 and SO-RB50 cells. After 48 h of transfection, cells were collected and treated with lysis buffer. The lysate was incubated with streptavidin beads pretreated with RNase-free Bovine Serum Albumin (BSA) and yeast tRNA. After incubation for 2 h, the RNA complex was eluted and isolated to detect the relative enrichment of PLCB1, MEF2C, and BMPR2 using qRT-PCR.

### Luciferase reporter gene assays

Luciferase reporter gene assays were conducted using a Dual-luciferase Reporter Assay System (Catalog number: E1910, Promega, Madison, WI, USA). Brief, the fragments of BMPR2 3′-UTR featuring a miR‐889-3p wild type (WT) or a mutated (MUT) binding site was cloned into a pmirGLO reporter vector (Catalog number: E1330, Promega), which were known as pmirGLO‐BMPR2‐WT or pmirGLO‐BMPR2‐MUT, respectively. They were then transfected using Lipofectamine 2000 into SO-RB50 and HXO-RB44 cells, which also co-expressed the miR-NC or miR-889-3p mimic. Forty-eight hours after transfection, the activity of firefly luciferase in each well was compared to that of Renilla luciferase.

### Xenograft assay

Ten male BALB/c nude mice were obtained from the Southern Medical University Laboratory Animal Centre at the age of six weeks and housed in a pathogen-free environment. The weight of the mice ranged from 18 to 20 g. The hospital Ethics Committee approved all animal experiments. Mice were randomly assigned to antagonist miR-889-3p and antagonist NC groups (n = 5 per group) prior to injection. To create animal models, HXO-RB44 cells were microinjected into the left armpit of mice, together with Antago miR-889-3p or Antago-NC obtained from GenePharma. Tumor development was tracked beginning on the fourth day, and the mice were weighed every 4 d. The formula used to determine the tumor size was volume (m^3^) = (length × width^2^) × 0.5. After 28 d, all mice were euthanized by CO_2_ inhalation and the tumor nodules were removed and weighed. The Animal Experimental Ethics Committee of Wuhan No. 1 Hospital approved the mouse experiments, and all animal experiments were performed in accordance with the ARRIVE guidelines.

### Statistical analysis

The GraphPad Prism 8.3.0 was used to analyze the data, and the results were given as mean ± SD. One-way ANOVA followed by Tukey’s post hoc test was used to assess differences across several groups, and *t*-tests were used to analyze differences between two groups. The expression levels of miR-889-3p and BMPR2 were compared using Pearson’s correlation analysis. Statistical significance was set at *P* < 0.05.

### Ethical approval

The medical ethics committee of Wuhan No. 1 Hospital approved this study (Wuhan, China). The availability of tissue sampling in experiment is in strict compliance and was in standardized on ethical ground by declaration of Helsiniki. Each patient signed a document requesting their informed permission. The animal tests were conducted out in Wuhan, all animal methods were carried out in accordance with ARRIVE principles and were authorized by the Animal Care Centre of Wuhan No. 1 Hospital.

### Participant agreement

Each patient completed an informed consent form in writing.

## Results

### MiR-889-3p modulates JNK/MAPK/ERK signaling in vitro to encourage Rb cell migration and proliferation

**Figure 1:**
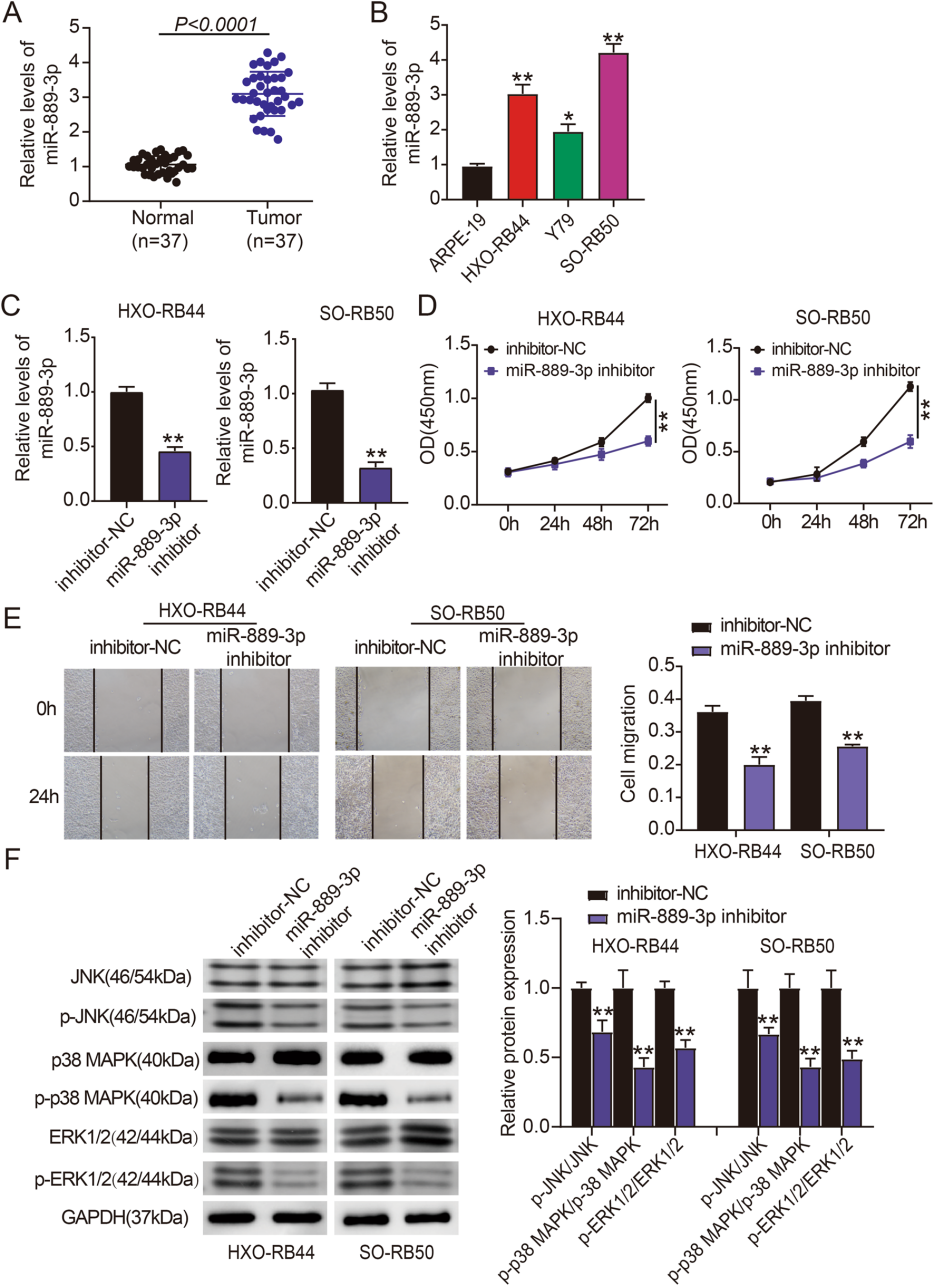
miR‐889-3p impacts retinoblastoma (Rb) cell migration and proliferation by modulating JNK/MAPK/ERK signaling pathway. (**A**) Quantitative reverse transcription PCR (qRT-PCR) was employed to assess the expression levels of miR-889-3p in Rb and matched neighboring normal tissues. (**B**) Using qRT-PCR, the relative expression of miR-889-3p in three Rb cell lines (SO-RB50, Y79, HXO-RB44) along with the normal human retinal epithelial cell line (ARPE-19) was examined, *P < 0.05, **P < 0.001 versus ARPE-19. (**C**) In HXO-RB44 and SO-RB50 cells, qRT-PCR was used to confirm the effectiveness of the miR-889-3p inhibitor’s knockdown, **P < 0.001 versus NC. (**D**) CCK-8 was performed to determine cell proliferation when miR-889-3p was down-expressed in HXO-RB44 and SO-RB50 cells, whereas **P < 0.001 versus NC. (**E**) A scratch test was employed to confirm cell migration after transfection with the miR-889-3p inhibitor or NC into HXO-RB44 and SO-RB50 cells, **P < 0.001 versus NC. (**F**) Western blot analysis of key molecules of JNK/MAPK/ERK signaling pathway in HXO-RB44 cells as well as SO-RB50 cells after miR-889-3p suppression, **P < 0.001 versus NC.

To determine whether miR-889-3p significantly contributes to illness, we first employed qRT-PCR to estimate the trend in miR-889-3p expression levels in Rb cells or tissues. Rb tissues, including SO-RB50, Y79, and HXO-RB44 Rb cells, expressed miR-889-3p at higher levels than matched nearby normal tissues or the ARPE-19 normal human retinal epithelial cell line (Fig. 1A,B). The biological function of miR-889-3p was determined using loss-of-function studies because HXO-RB44 and SO-RB50 cell lines expressed miR-889-3p at higher levels. The downregulated expression of miR-889-3p was confirmed by qRT-PCR (Fig. 1C). The miR-889-3p inhibitor dramatically reduced the viability of HXO-RB44 and SO-RB50 cells, according to the CCK-8 assay (Fig. 1D). Scratch testing revealed that HXO-RB44 and SO-RB50 cells treated with the miR-889-3p inhibitor migrated considerably less than those in the NC group (Fig. 1E). Additionally, we assessed the key proteins of the JNK/MAPK/ERK signaling pathway by western blotting and found that JNK, p38 MAPK, and ERK1/2 were less phosphorylated when miR-889-3p was downregulated (Fig. 1F). These findings indicate that miR-889-3p modulates JNK/MAPK/ERK signaling in vitro to promote the proliferation and migration of Rb cells.

### miR‐889-3p knockdown inhibits Rb tumor growth in vivo

**Figure 2:**
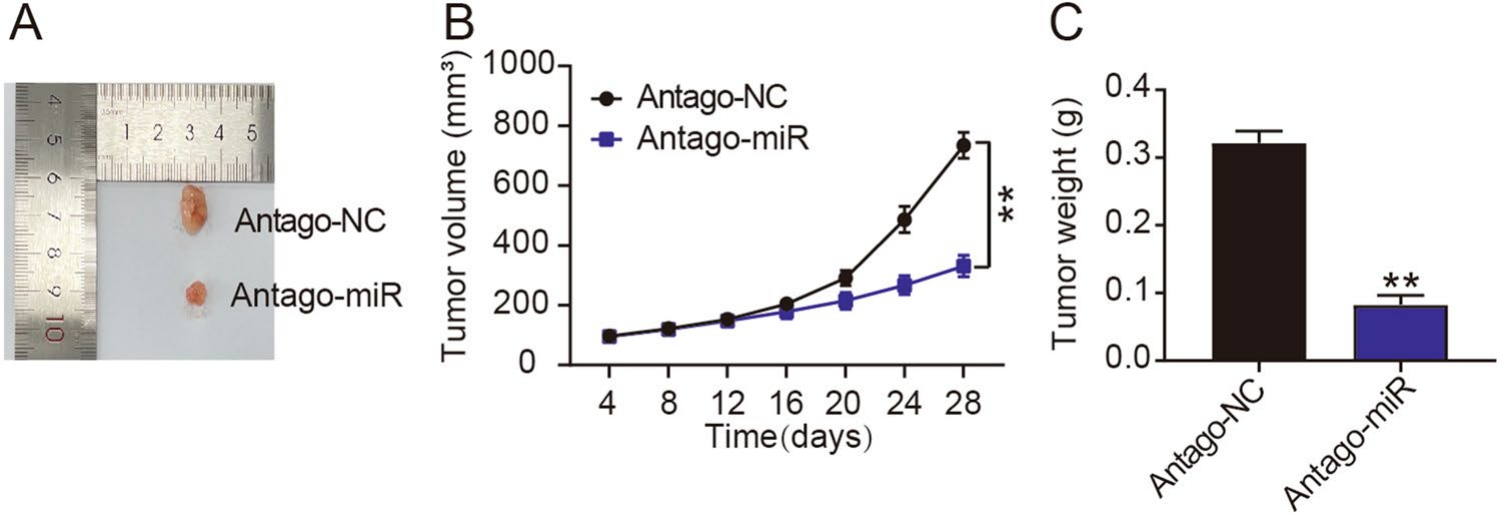
In vivo, miR-889-3p supports the formation of retinoblastoma (Rb) tumors. (**A**) The tumor samples from the nude mice given Antago-NC or Antago-miR-889-3p (Antago-miR) injections are shown here. (**B**) The curve of tumor volume from the nude mice injected with Antago-NC or Antago-miR, whereas **P < 0.001 versus NC. (**C**) The tumor weight of the nude mice was measured on the 28th day after injection with Antago-NC or Antago-miR, **P < 0.001 versus NC.

The effect of miR-889-3p on Rb cell tumorigenicity in vivo was examined. Compared with the Antago-NC group, the Antago miR-889-3p group showed a significant reduction in tumor growth (Fig. 2A), volume (Fig. 2B), and weight (Fig. 2C). Overall, our results imply that miR-889-3p knockdown inhibits Rb cell tumor formation in vivo.

### Directly targeting BMPR2 by miR889-3p

**Figure 3:**
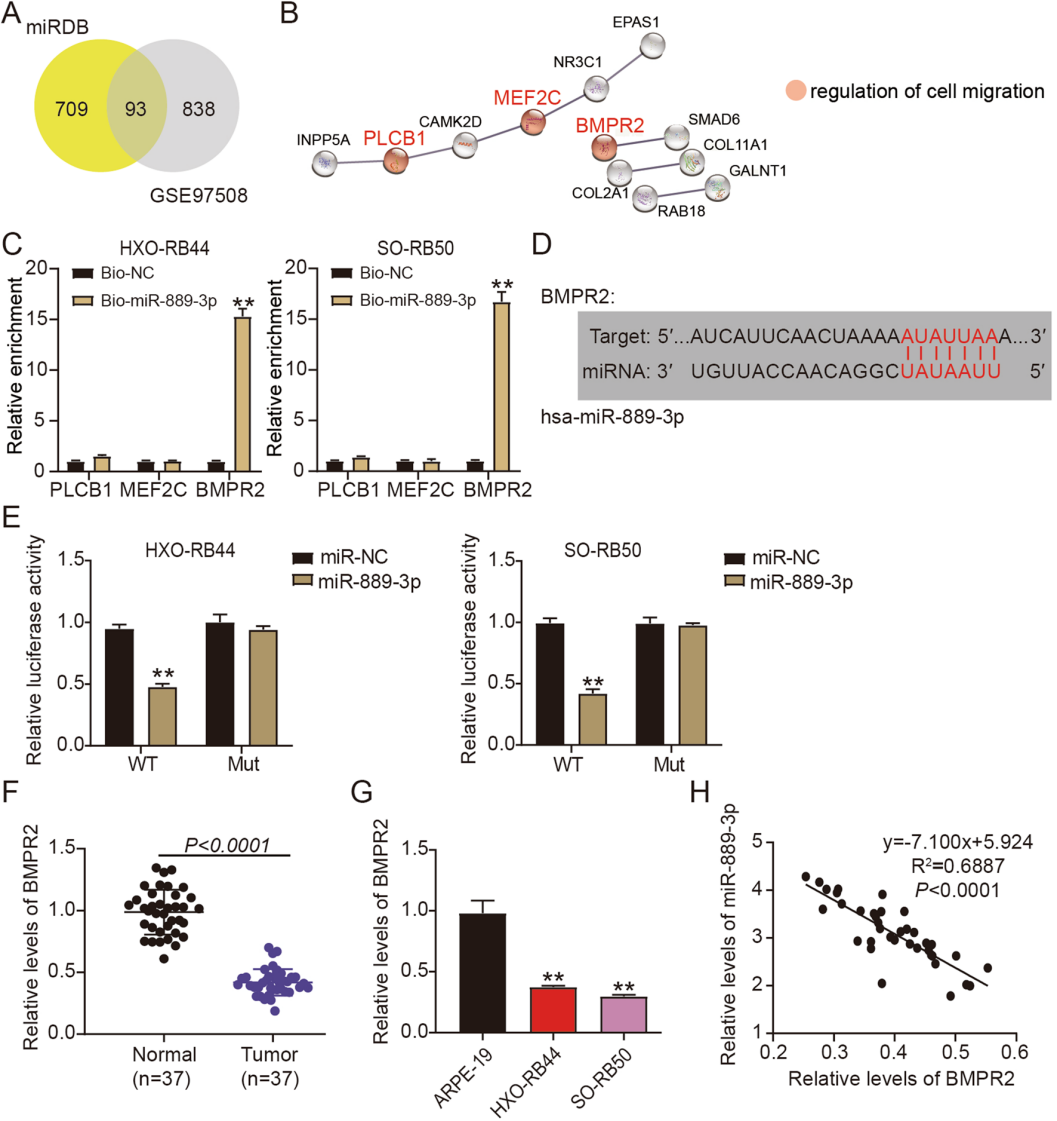
miR‐889-3p directly targets BMPR2. (**A**) Ninety-three genes overlapped with miRDB and GSE97508 according to the Venny 2.1. (**B**) Gene Ontology (GO) enrichment of 93 genes using STRING software. (**C**) The relative enrichment of PLCB1, MEF2C, and BMPR2 in Rb cells transfected with Bio-NC or Bio-miR-889-3p was assessed by an RNA pull-down assay. **P < 0.001 versus Bio-NC. (**D**) The binding site in the 3′UTR of BMPR2 for miR‐889-3p was predicted by TargetScan. (**E**) Luciferase activity was evaluated in HXO-RB44 and SO-RB50 cells co-transfected with miR‐889-3p mimics or miRNA negative control and wild type (WT) or mutant (MUT) 3′UTR of BMPR2, **P < 0.001 versus miR-NC. (**F**) BMPR2 expression was determined by qRT-PCR in Rb tissues and matched with nearby normal tissues. (**G**) In two Rb cell lines and a normal retinal epithelial cell line (ARPE-19), BMPR2 expression was determined by qRT-PCR (HXO-RB44 and SO-RB50); **P < 0.001 versus ARPE-19 cells. (**H**) MiR-889-3p and BMPR2 were used in Pearson’s association analysis.

We predicted a possible target for the miR-889-3p molecule using the miRNA target analysis tool miRDB and found downregulated genes in Rb samples using an mRNA microarray GSE97508 from GEO DataSets (screening criteria: adj. *P* < 0.05 and logFC <  − 2) to gain a better understanding of the molecular process behind the capacity of miR-889-3p to promote Rb progression. Venny 2.1 analysis showed that 93 genes overlapped with miRDB and GSE97508 (Fig. 3A). We then uploaded these 93 genes to STRING for Gene Ontology (GO) enrichment, which showed that PLCB1, MEF2C, and BMPR2 were correlated with the regulation of cell migration (Fig. 3B). After performing the RNA pull-down assay, only BMPR2 was pulled down by miR-889-3p in R cells (Fig. 3C). Figure 3D shows that miR-889-3p potentially targets *BMPR2* mRNA. To further validate BMPR2 as a target of miR-889-3p, we performed luciferase reporter gene assays using luciferase production plasmids expressing either WT or MUT BMPR2. According to these findings, miR-889-3p mimic transfection in HXO-RB44 and SO-RB50 cells caused the BMPR2-WT construct, but not the BMPR2-MUT construct, to decrease luciferase activity (Fig. 3E). We verified that BMPR2 is a downstream target of miR-889-3p before examining its expression in Rb tissues and cells. The results demonstrated that *BMPR2* mRNA levels in Rb tissues or cells, such as SO-RB50 and HXO-RB44, were considerably lower than those in matched normal tissues (Fig. 3F) or in the normal human retinal epithelial cell line, ARPE-19 (Fig. 3G). Furthermore, miR-889-3p expression was inversely associated with BMPR2 expression (Fig. 3H). These findings indicate that miR-889-3p specifically targets BMPR2.

### Knockdown of BMPR2 blocked the miR-889-3p inhibitory impacts on activation, cell proliferation, and migration of JNK/MAPK/ERK signaling in Rb cells

**Figure 4:**
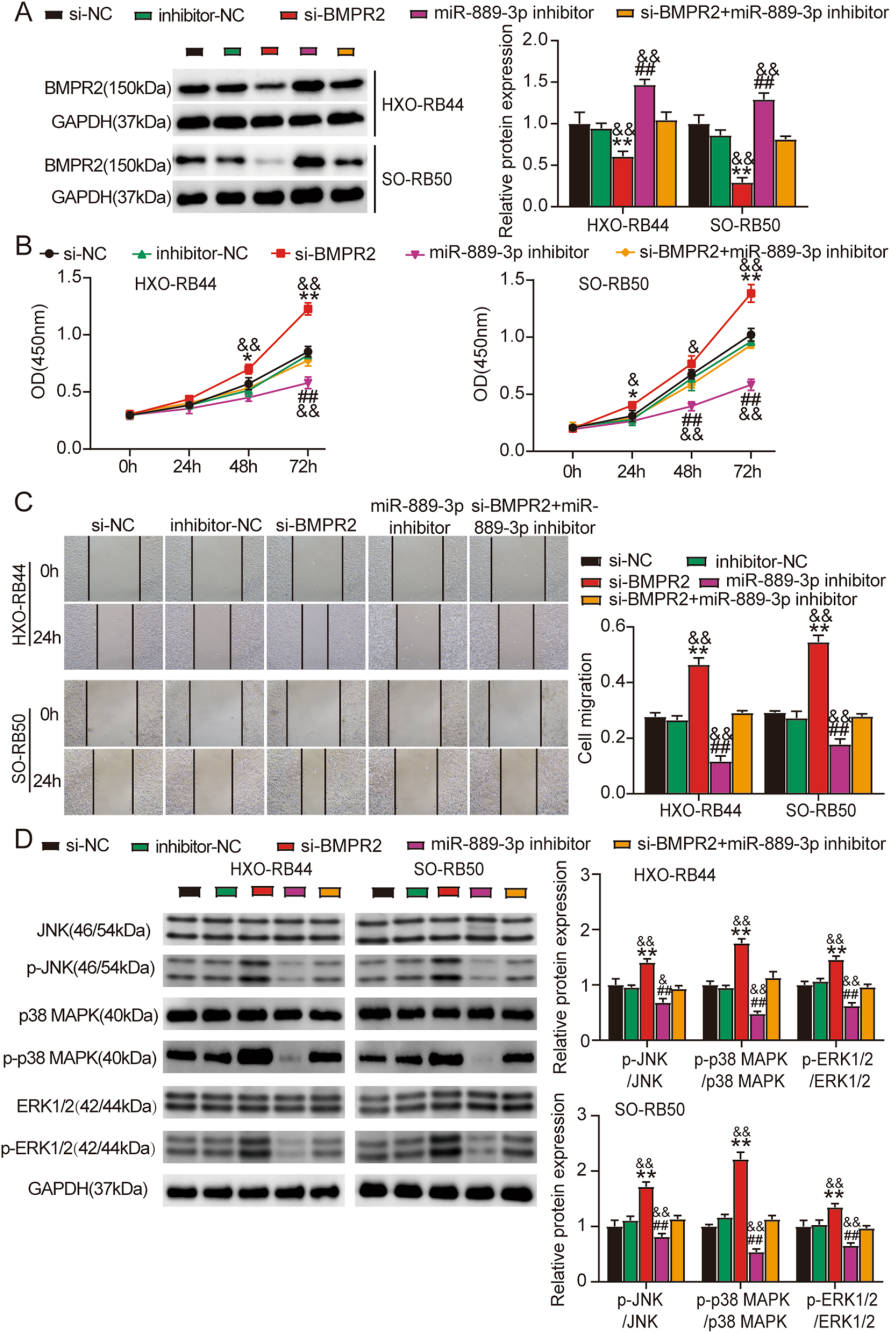
BMPR2 inhibited the biological effects of miR-889-3p in Rb cells. (**A**) BMPR2 expression level was determined using western blotting in HXO-RB44 and SO-RB50 cells transfected with si-NC, inhibitor-NC, si-BMPR2, miR-889-3p inhibitor or co-transfection of si-BMPR2 and miR-889-3p inhibitors. (**B**–**D**) A CCK-8 assay (**B**), scratch test (**C**), as well as western blotting (**D**) were utilized to compare the effect of co-transfection of miR-889-3p inhibitor and si-BMPR2 on cell proliferation, migration, and expression of key molecules of the JNK/MAPK/ERK signaling pathway in HXO-RB44, as well as in SO-RB50 cells transfected with the miR-889-3p inhibitor or si-BMPR2. ##*P* < 0.001 versus inhibitor-NC; *P < 0.05, **P < 0.001 versus si-NC; &*P* < 0.05, &&*P* < 0.001 versus si-BMPR2 + miR-889-3p inhibitor.

We performed rescue trials to investigate whether miR-889-3p exerted its biological function by regulating BMPR2 expression. The protein expression of BMPR2 was found to be lower in SO-RB50 and HXO-RB44 cells transfected with si-BMPR2, indicating that the BMPR2 interference fragment was valid. Compared to inhibitor-NC, BMPR2 expression at the protein level was elevated in HXO-RB44 and SO-RB50 cells transfected with the miR-889-3p inhibitor (Fig. 4A). As expected, cell viability (Fig. 4B), migration (Fig. 4C), and protein phosphorylation of JNK, p38 MAPK, and ERK1/2 (Fig. 4D) were diminished in SO-RB50 and HXO-RB44 cells transfected with the miR-889-3p inhibitor compared to inhibitor-NC, whereas si-BMPR2 had the opposite biological functions of the miR-889-3p inhibitor. Additionally, the effects of the miR-889-3p inhibitor were reduced by cotransfection with si-BMPR2 and miR-889-3p inhibitors. These findings suggest that miR-889-3p promotes Rb migration, activation, and proliferation in JNK/MAPK/ERK signaling by negatively regulating BMPR2 expression.

## Discussion

Rb is the most dangerous intraocular tumor of the retina in children, and its incidence is increasing worldwide^23^. According to a growing body of evidence, miRNAs are essential players in the pathophysiology of Rb^10^. As a result, miRNAs are considered potent molecular indicators for both diagnosis and prognosis as well as treatment targets in Rb. Here, we found that miR-889-3p was upregulated in Rb and that silencing miR-889-3p suppressed the malignancy of Rb cells by targeting BMPR2 to activate the JNK/MAPK/ERK pathway.

Previous studies have demonstrated that miR-889-3p functions in a variety of solid tumors. For instance, in cervical cancer, decreased miR-889-3p expression promoted cell proliferation, motility, and invasion while suppressing apoptosis and radiosensitivity^12,13^. However, according to another study^16^, miR-889-3p functions as an oncogene in osteosarcoma^16^. Furthermore, miR-889-3p promoted the invasive capacity of malignant peripheral nerve sheath tumors^15^. The function of miR-889-3p in Rb currently remains unknown. In the current study, we found that Rb tissues and cell lines exhibited significantly higher levels of miR-889-3p. MiR-889-3p can be knocked down to inhibit tumor growth, migration, and proliferation. Our findings are in accordance with those of previous research on malignant peripheral nerve sheath tumors and osteosarcoma, suggesting that miR-889-3p is an oncogene in Rb.

There is growing evidence that miRNAs may bind to target genes’ 3-UTRs and change their expression^6^. MiR-889-3p has been shown to affect multiple genes involved in the tumorigenesis of several malignancies^13–16^. Here, we discovered that miR-889-new 3p’s target gene was BMPR2. A crucial component of BMP signaling is BMPR2, a member of the BMP receptor family of transmembrane serine/threonine kinases^24^. Recently, abnormal BMPR2 expression was identified in lung cancer^17^, pancreatic ductal adenocarcinoma^18^, and osteosarcoma^19^. However, the role of BMPR2 in the development ofRb remains unclear. The results of this study demonstrated an inverse link between miR-889-3p and BMPR2 expression in Rb tumors, and a luciferase reporter assay further demonstrated a direct binding relationship. Furthermore, the restraint of BMPR2 had inverse biological effects against the hindrance of miR-889-3p, indicating that BMPR2 knockdown promoted the migration, proliferation, and invasion of Rb cells.

The JNK/MAPK/ERK signaling pathway is a key pathway related to multiple human diseases, including osteoarthritis^22^, gastric cancer^25^, and obesity^26^. In cancer, the activation of the JNK/MAPK/ERK signaling pathway by PAGE4 promotes prostate cancer cell survival^27^. According to the previous study from Xiao et al.^22^, BMPR2 overexpression inhibits the activation of the JNK/MAPK/ERK signaling pathway in osteoarthritis. In our study, the downregulation of BMPR2 enhanced the phosphorylation levels of JNK, p38 MAPK, and ERK1/2 in Rb cells, suggesting that BMPR2 knockdown activated the JNK/MAPK/ERK signaling pathway in Rb cells. Our results are in agreement with those reported by Xiao et al. in osteoarthritis.

Despite these limitations, we determined the role and regulation mechanism in Rb cells of miR-889-3p. The number of clinical samples was insufficient to determine the clinical value of miR-889-3p; therefore, more clinical samples should be collected in the future to determine the function of miR-889-3p. Additionally, the upstream role of miR-889-3p in Rb has not yet been investigated. In the future, we will investigate the important regulators that improve the miR-889-3p regulatory network in Rb.

## Conclusion

These results demonstrate that miR-889-3p targets BMPR2 and activates the JNK/MAPK/ERK signaling pathway, promoting the growth and metastasis of Rb tumors. The discovery of a new miR-889-3p/BMPR2 regulatory axis may provide a new therapeutic target for Rb (Supplementary information).

### Supplementary Information

Supplementary Information.

## Data Availability

All of the information produced or analysed for this inquiry is contained in this article.
