# Supplementary figures and images for: miR-889-3p targeting BMPR2 promotes the development of retinoblastoma via JNK/MAPK/ERK signaling
Source: Sci Rep. 2024 Mar 27;14:7277. doi: 10.1038/s41598-023-49994-2 (PMC10973389; doi:10.1038/s41598-023-49994-2)

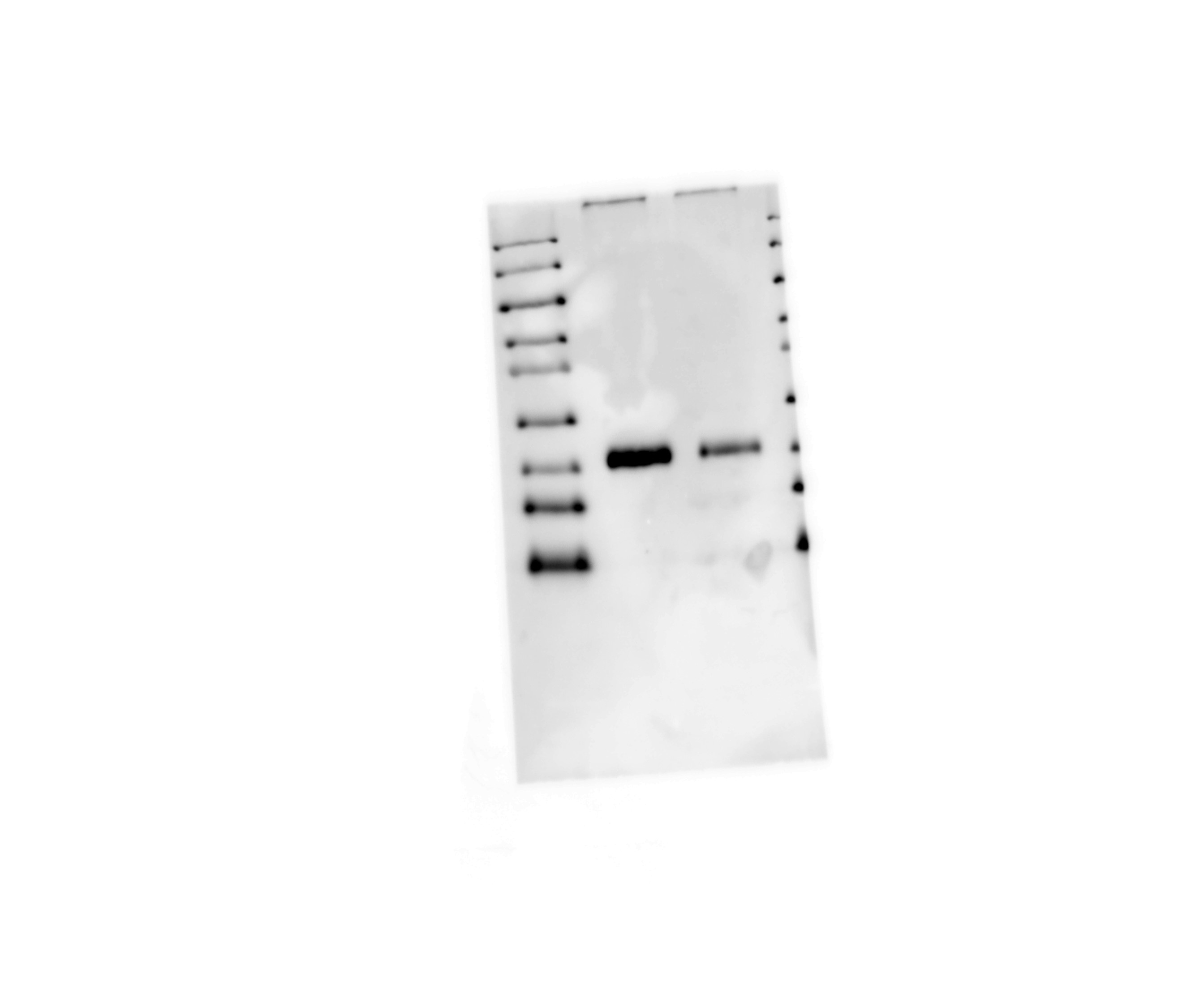

Supplement: Supplementary file 1 — Supplementary Information. [file 41598_2023_49994_MOESM1_ESM.zip › Fig 1F HXO-RB44 p-p38MAPK.jpg]

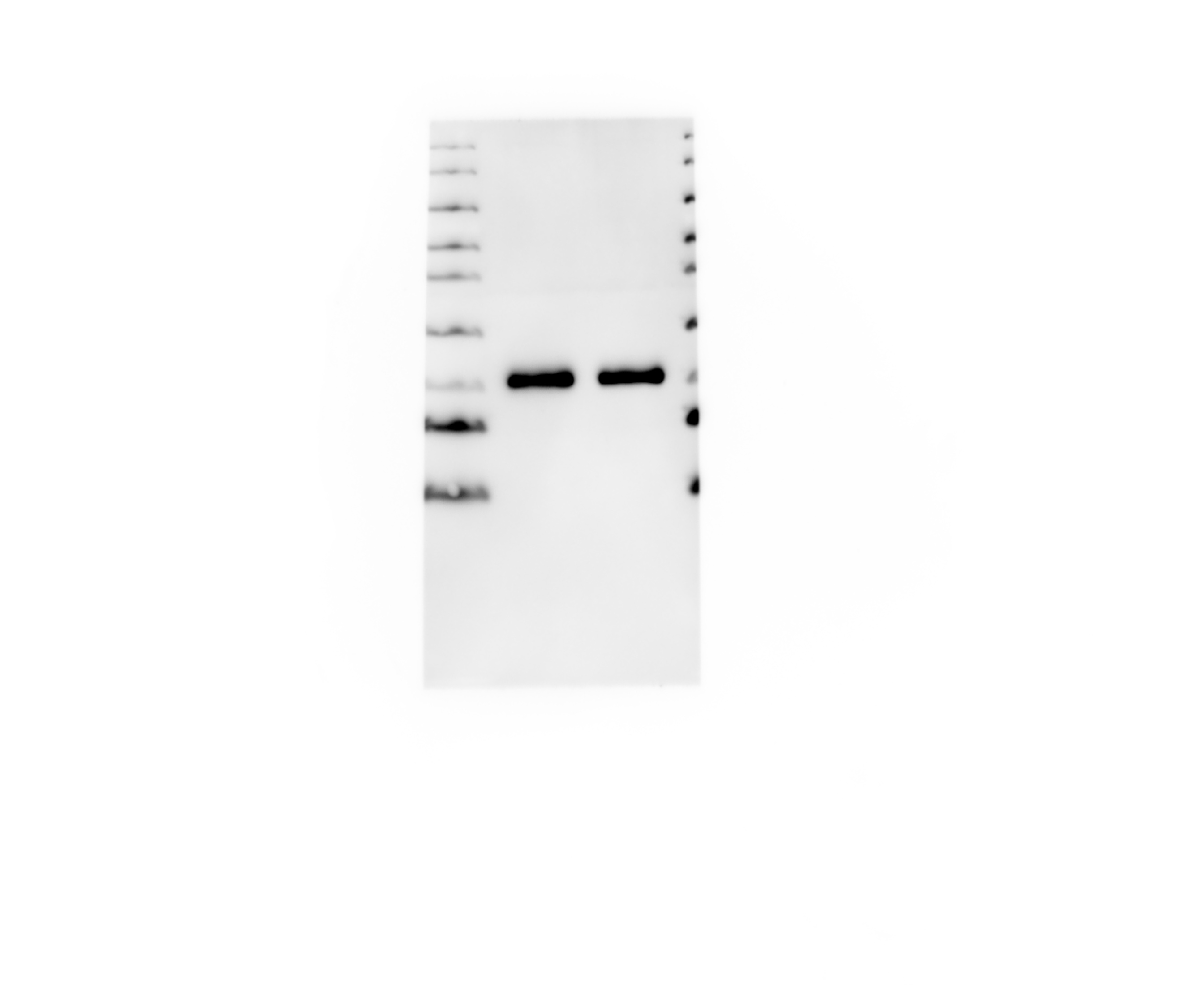

Supplement: Supplementary file 1 — Supplementary Information. [file 41598_2023_49994_MOESM1_ESM.zip › Fig 1F SO-RB50 GAPDH.jpg]

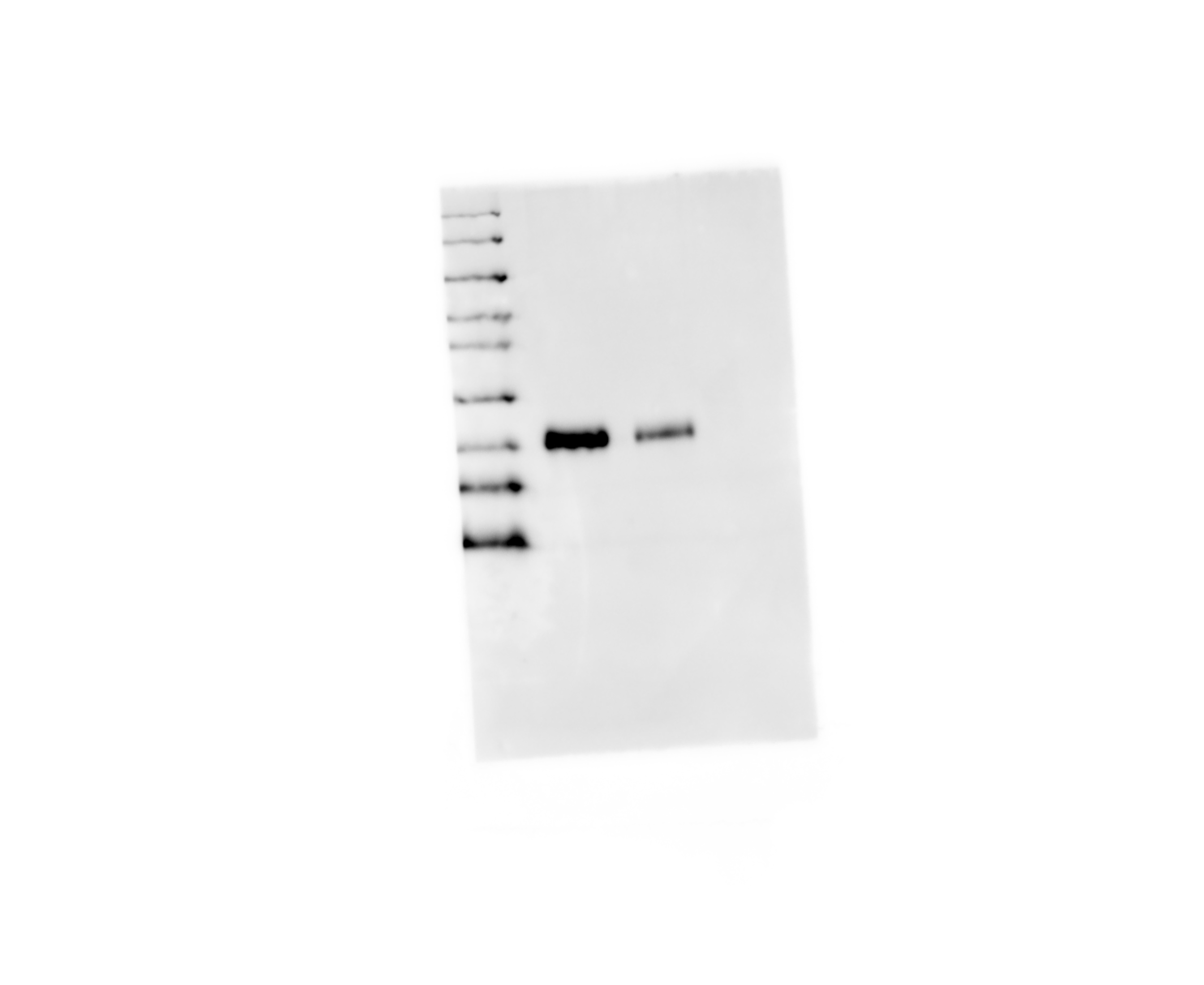

Supplement: Supplementary file 1 — Supplementary Information. [file 41598_2023_49994_MOESM1_ESM.zip › Fig 1F SO-RB50 p-p38MAPK.jpg]

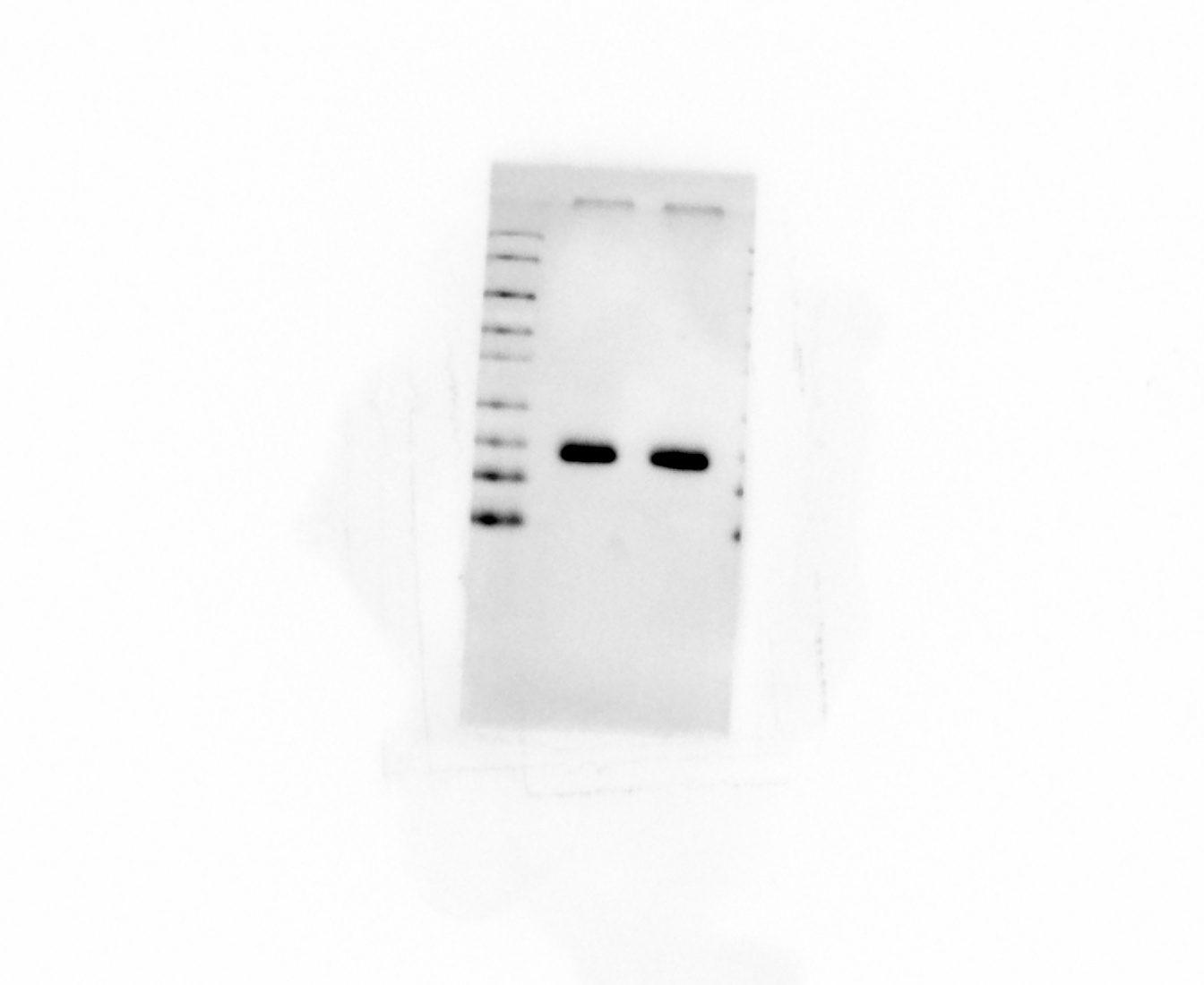

Supplement: Supplementary file 1 — Supplementary Information. [file 41598_2023_49994_MOESM1_ESM.zip › Fig 1F SO-RB50 p38MAPK.jpg]

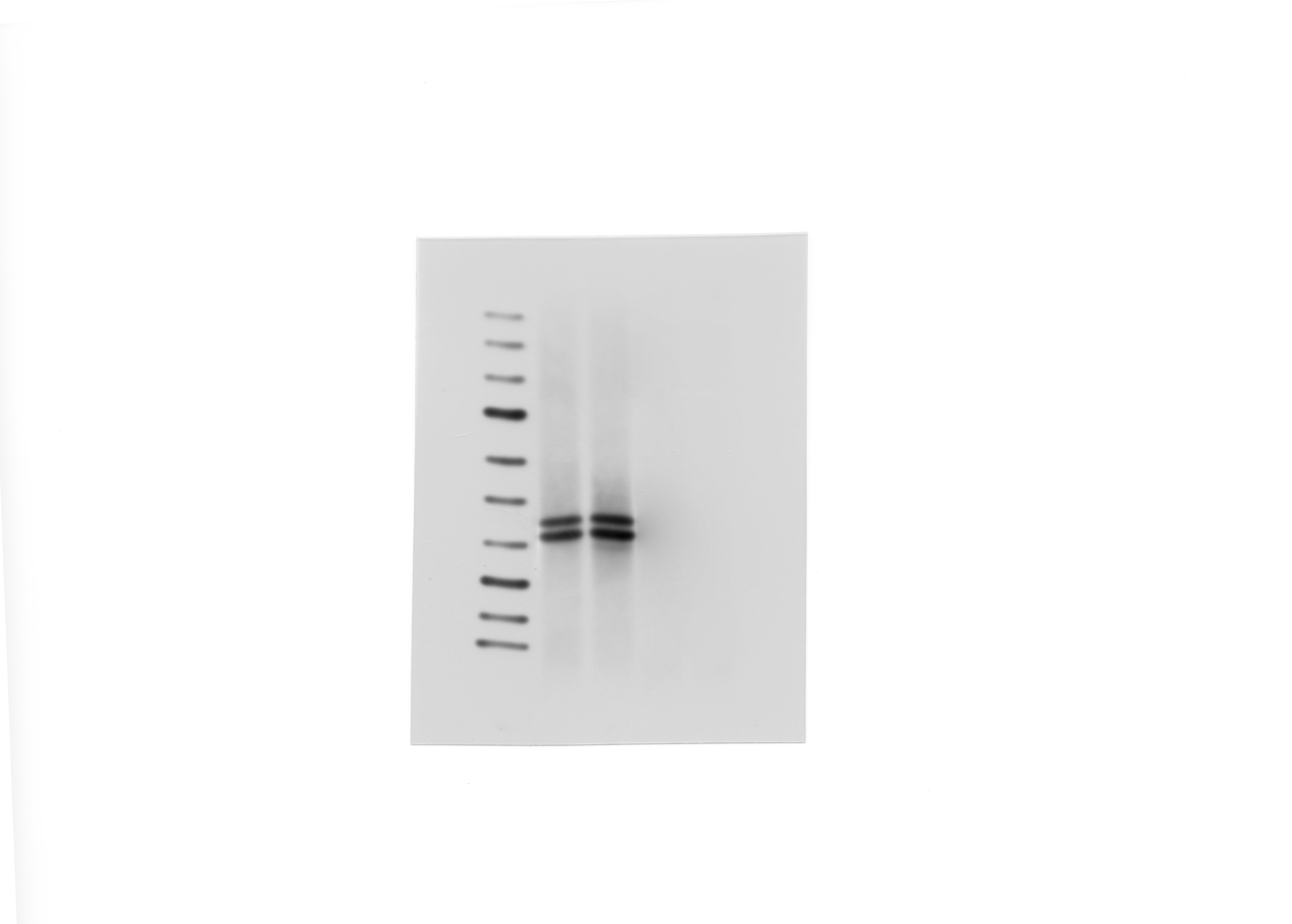

Supplement: Supplementary file 1 — Supplementary Information. [file 41598_2023_49994_MOESM1_ESM.zip › Fig 1F SO-RB50 ERK.jpg]

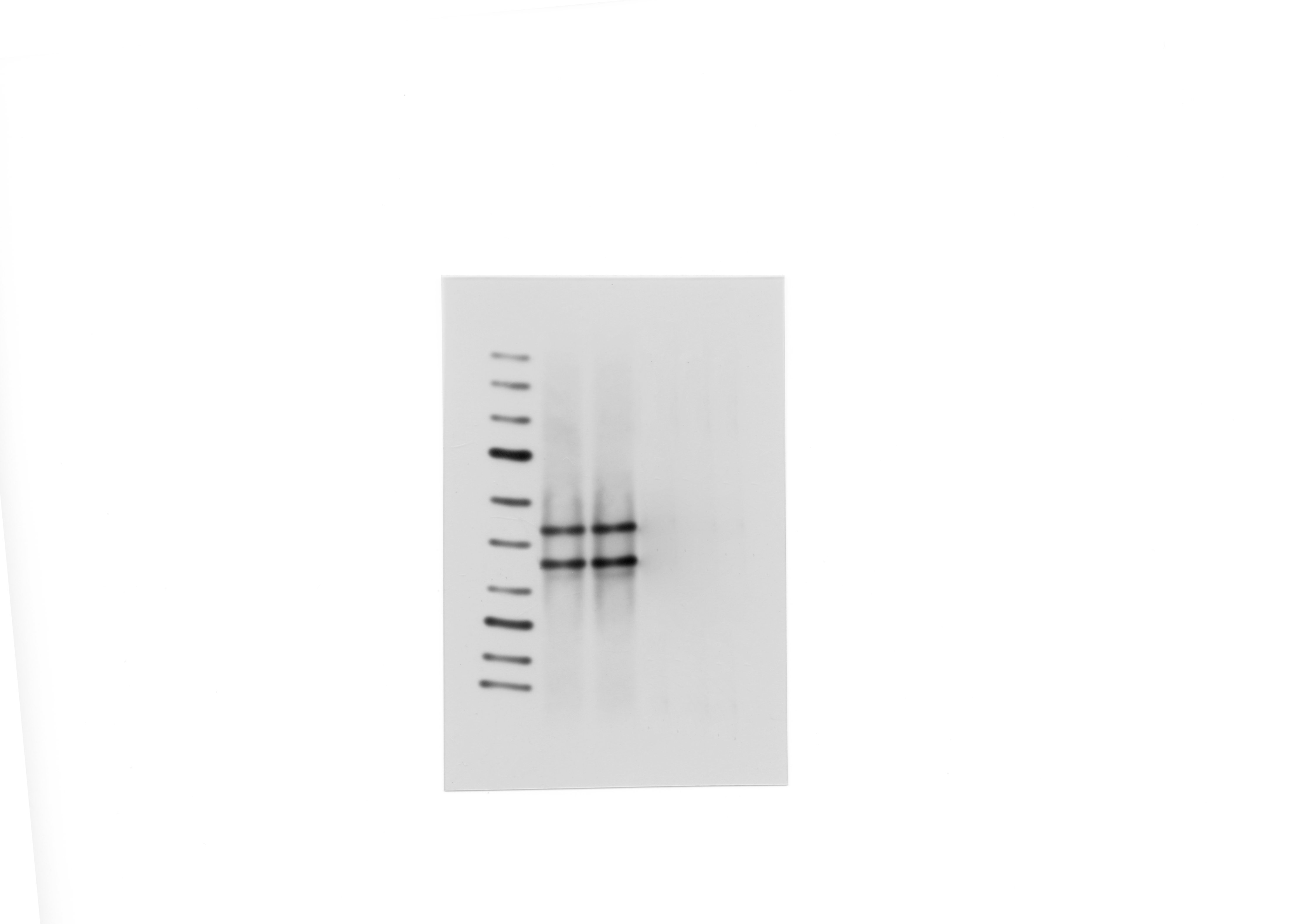

Supplement: Supplementary file 1 — Supplementary Information. [file 41598_2023_49994_MOESM1_ESM.zip › Fig 1F SO-RB50 JNK.jpg]

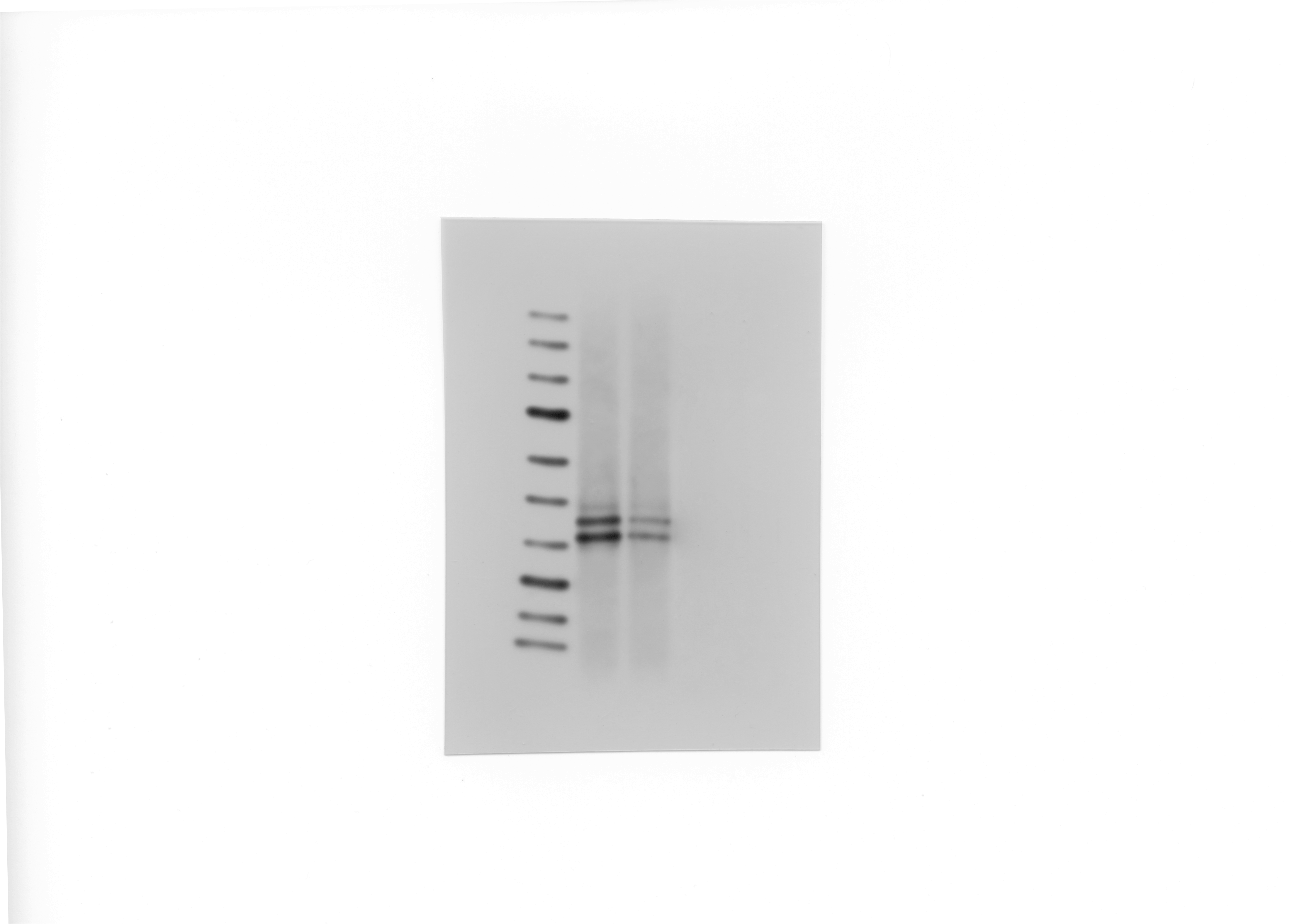

Supplement: Supplementary file 1 — Supplementary Information. [file 41598_2023_49994_MOESM1_ESM.zip › Fig 1F SO-RB50 p-ERK.jpg]

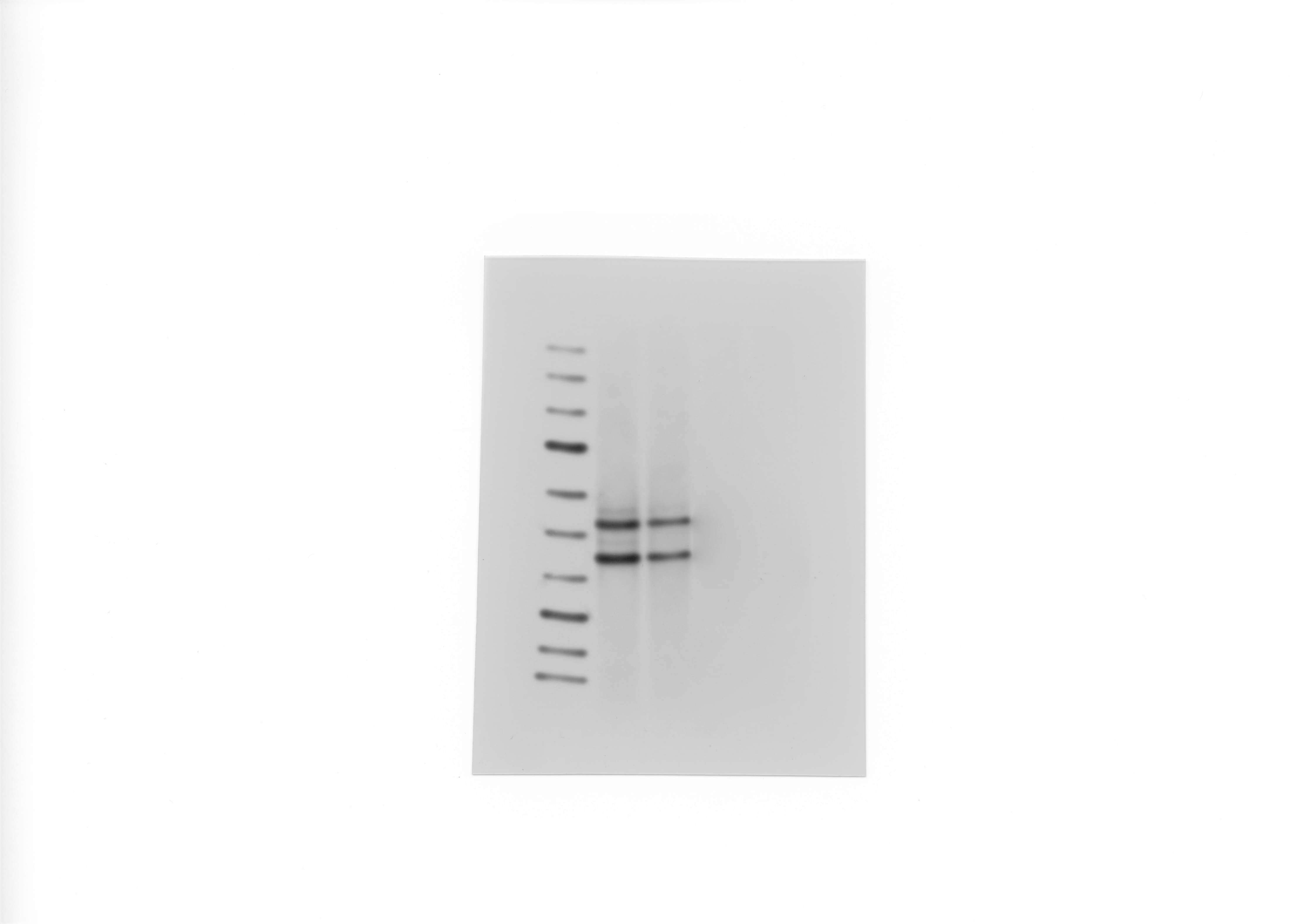

Supplement: Supplementary file 1 — Supplementary Information. [file 41598_2023_49994_MOESM1_ESM.zip › Fig 1F SO-RB50 p-JNK.jpg]

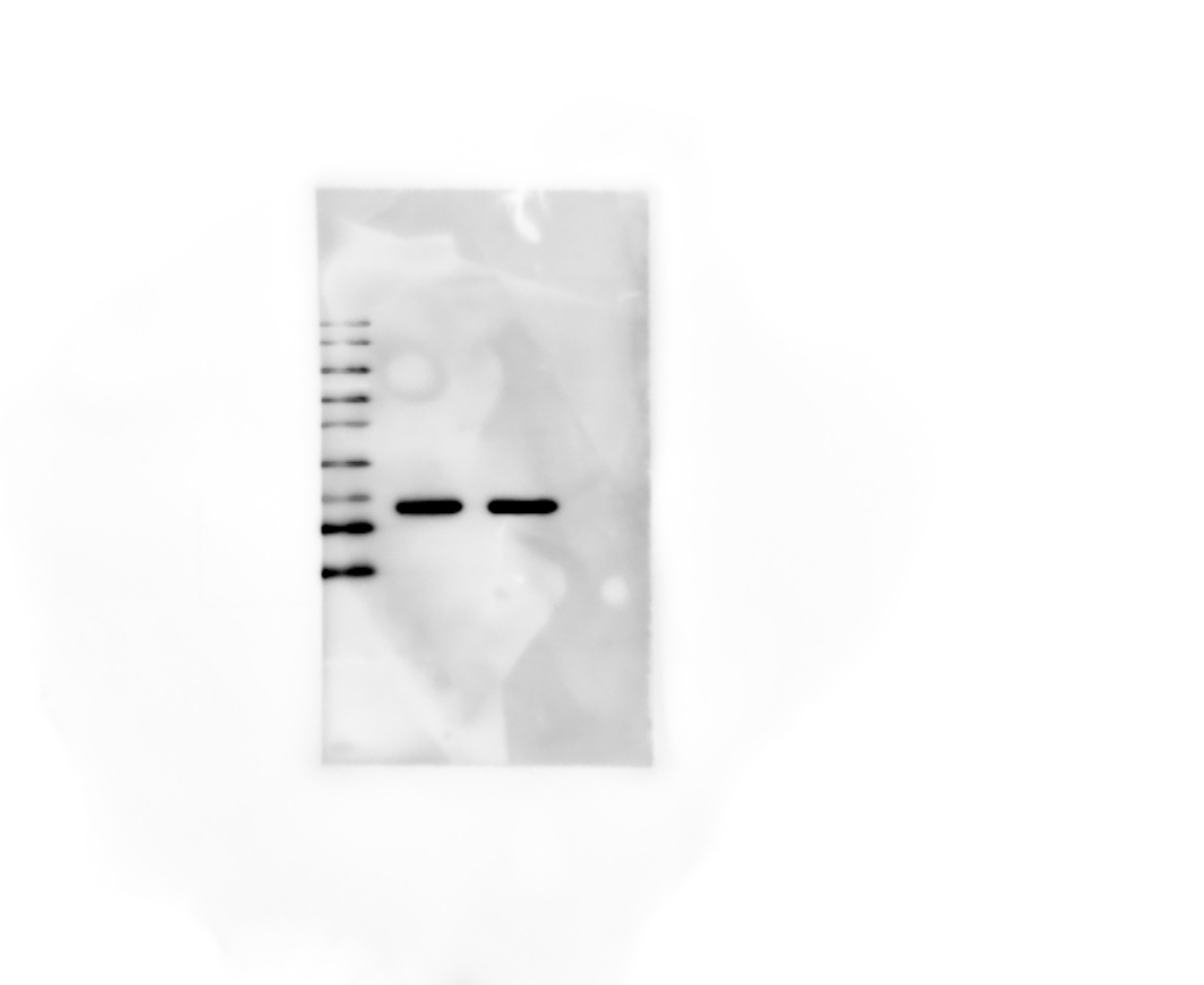

Supplement: Supplementary file 1 — Supplementary Information. [file 41598_2023_49994_MOESM1_ESM.zip › Fig1F HXO-RB44 GAPDH.jpg]

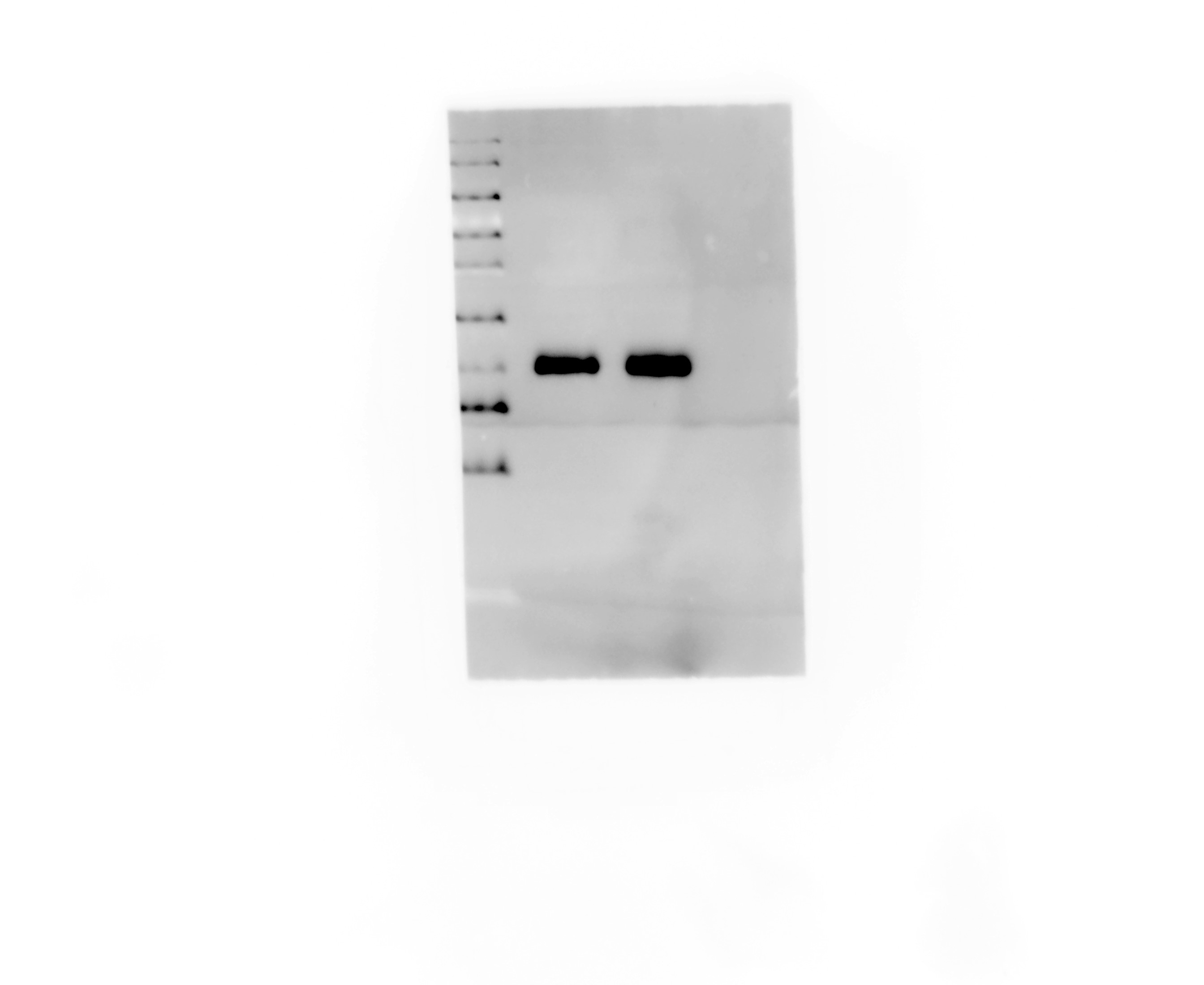

Supplement: Supplementary file 1 — Supplementary Information. [file 41598_2023_49994_MOESM1_ESM.zip › Fig1F HXO-RB44 p38MAPK.jpg]

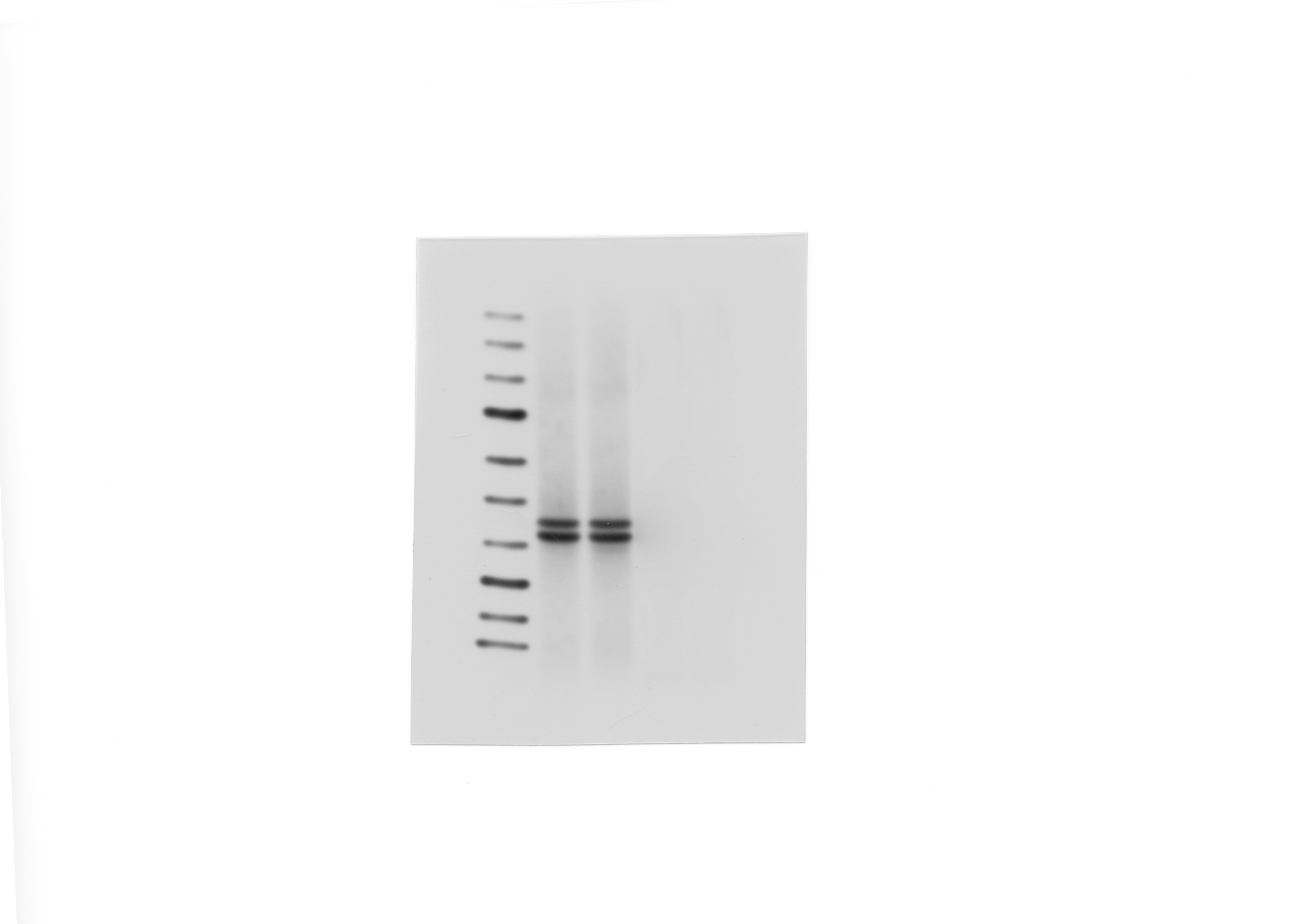

Supplement: Supplementary file 1 — Supplementary Information. [file 41598_2023_49994_MOESM1_ESM.zip › Fig1F HXO-RB44 ERK.jpg]

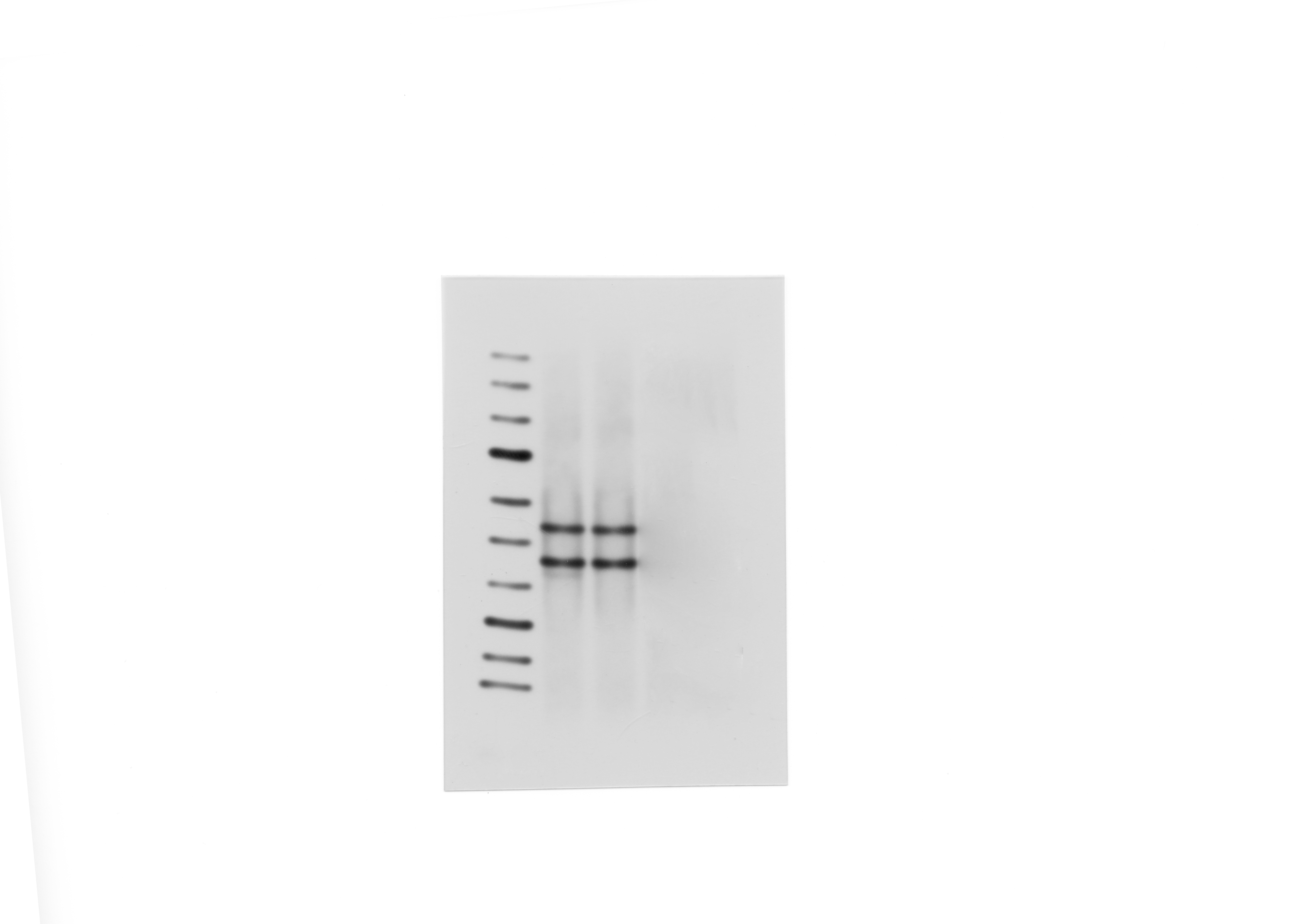

Supplement: Supplementary file 1 — Supplementary Information. [file 41598_2023_49994_MOESM1_ESM.zip › Fig1F HXO-RB44 JNK.jpg]

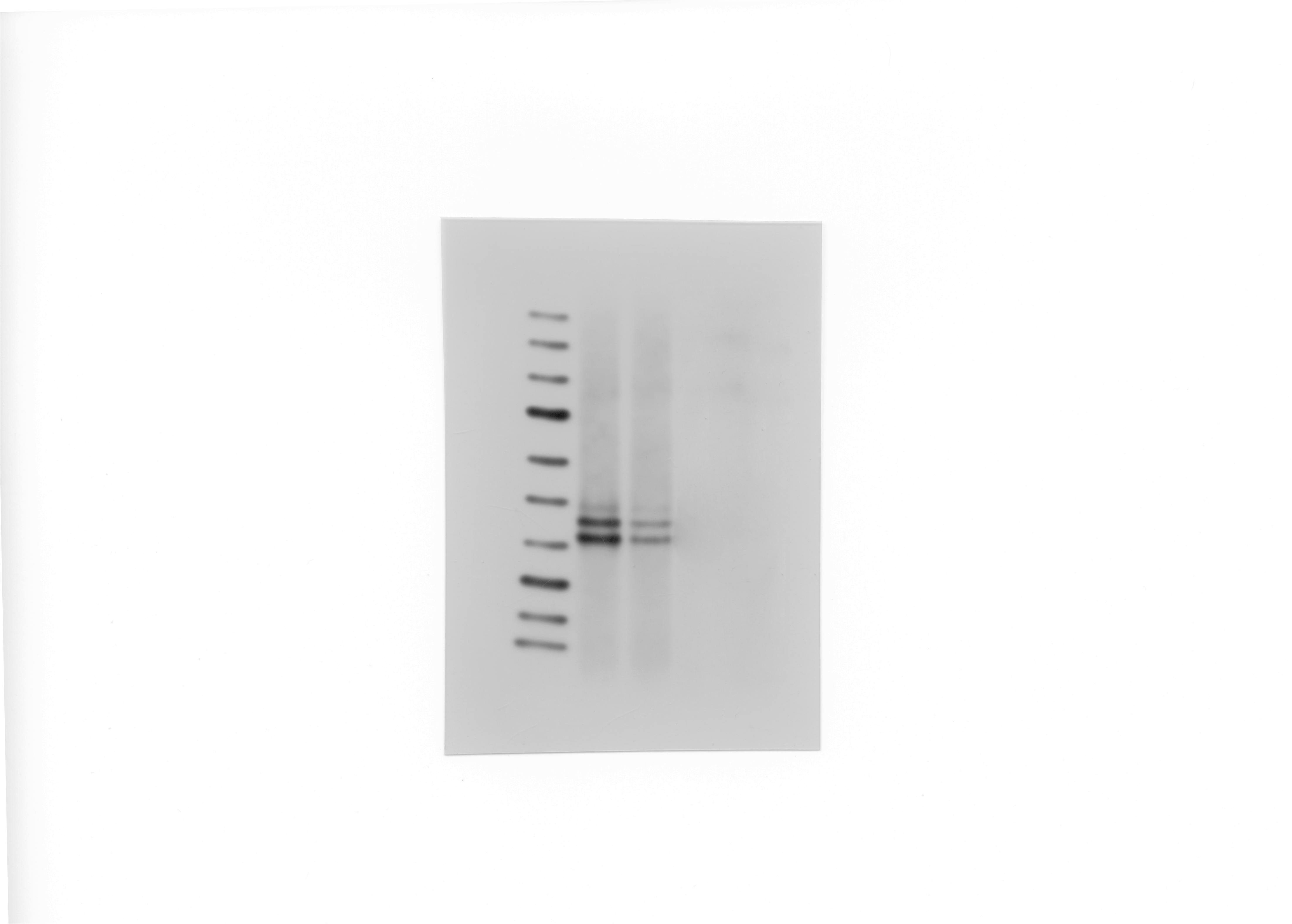

Supplement: Supplementary file 1 — Supplementary Information. [file 41598_2023_49994_MOESM1_ESM.zip › Fig1F HXO-RB44 p-ERK.jpg]

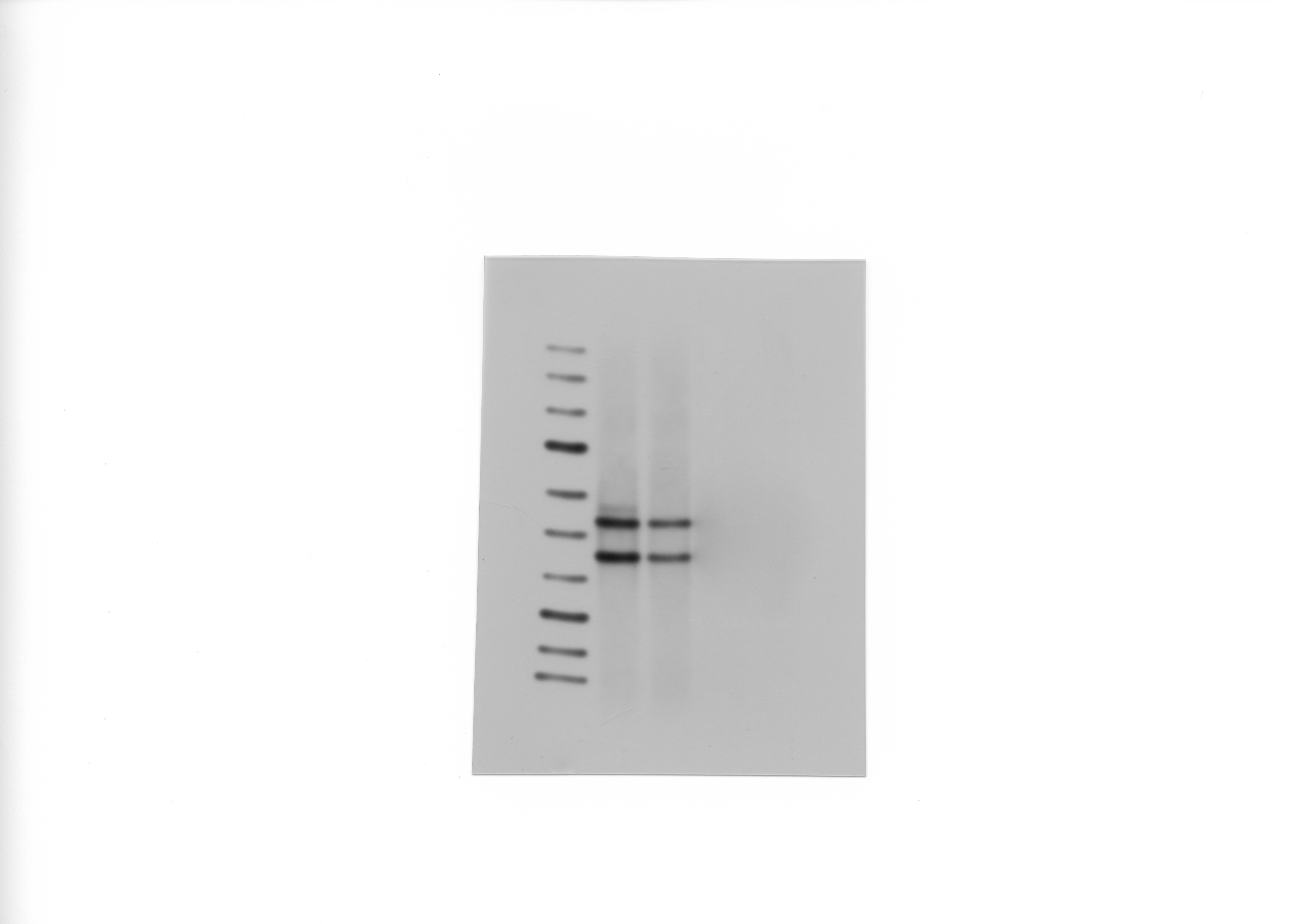

Supplement: Supplementary file 1 — Supplementary Information. [file 41598_2023_49994_MOESM1_ESM.zip › Fig1F HXO-RB44 p-JNK.jpg]

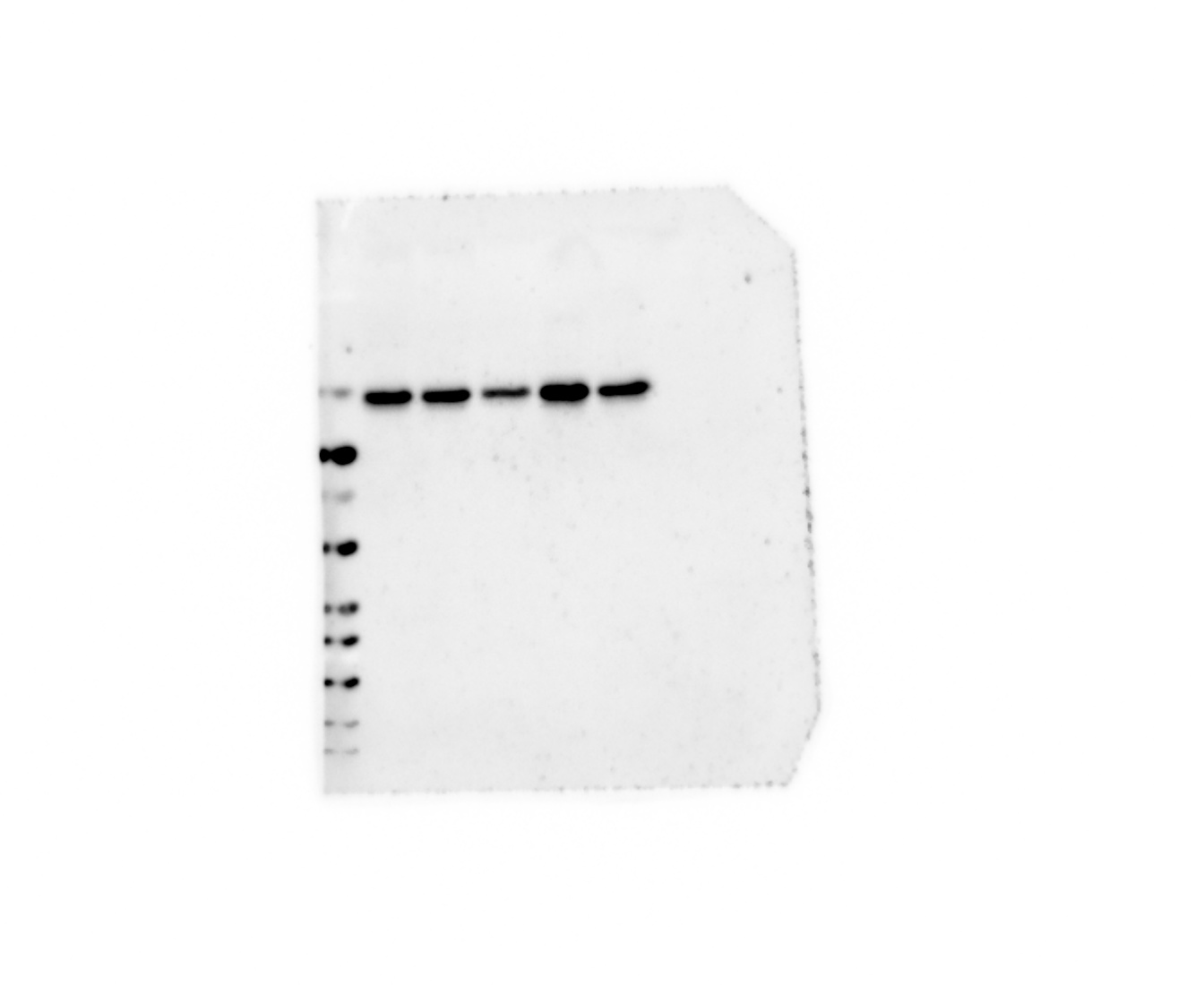

Supplement: Supplementary file 1 — Supplementary Information. [file 41598_2023_49994_MOESM1_ESM.zip › Fig4A HXO-RB44 BMPR2.jpg]

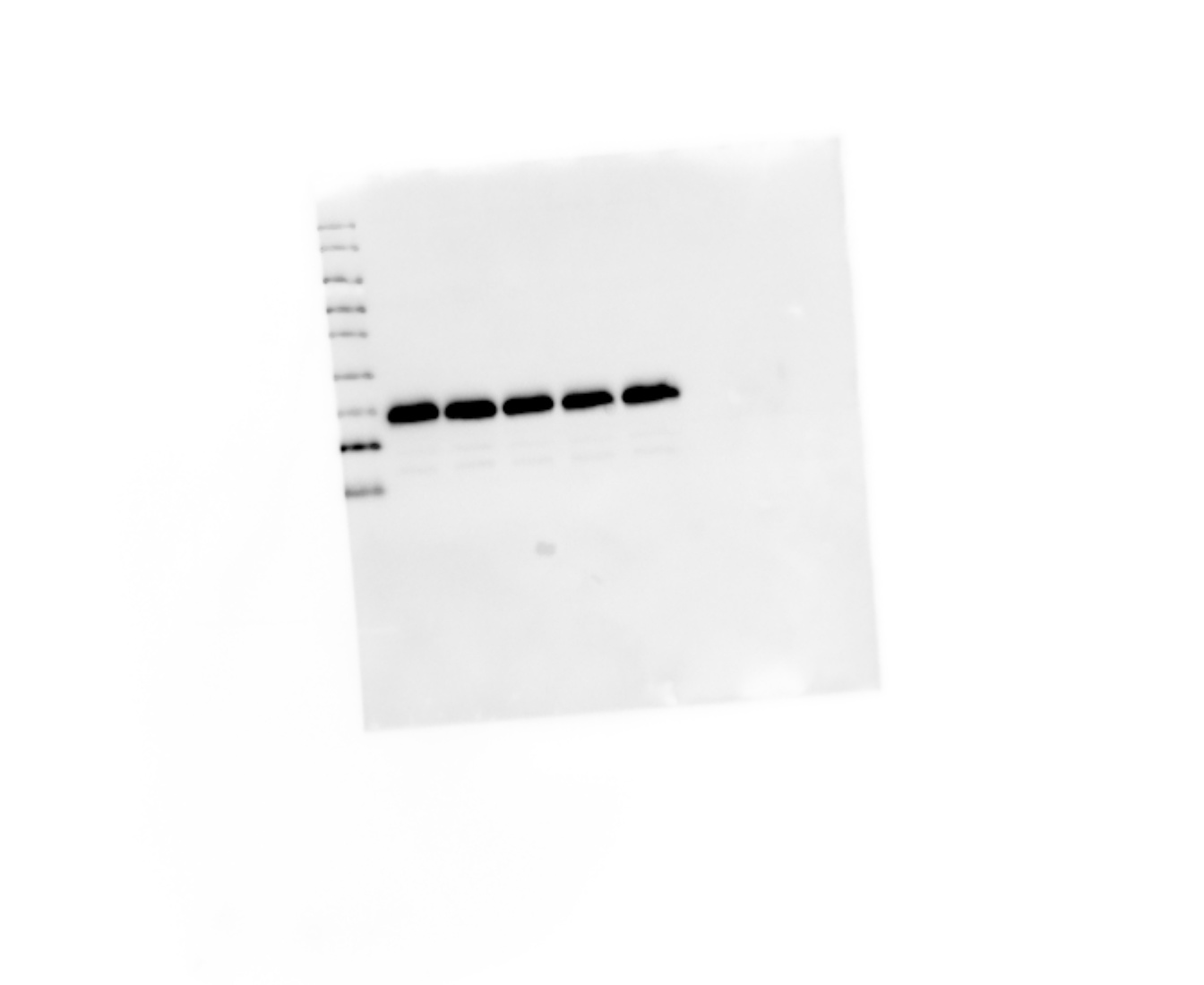

Supplement: Supplementary file 1 — Supplementary Information. [file 41598_2023_49994_MOESM1_ESM.zip › Fig4A HXO-RB44 GAPDH.jpg]

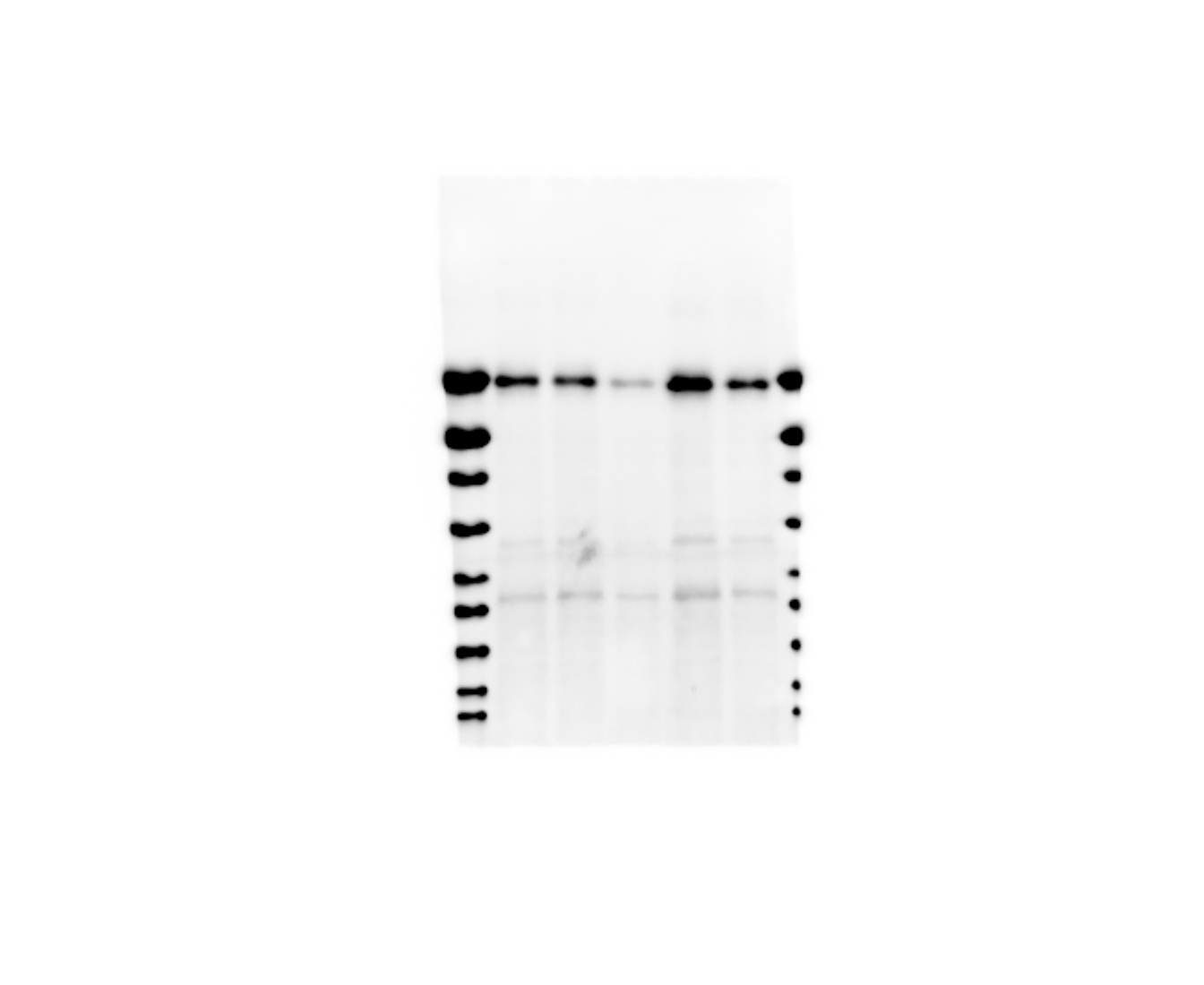

Supplement: Supplementary file 1 — Supplementary Information. [file 41598_2023_49994_MOESM1_ESM.zip › Fig4A SO-RB50 BMPR2.jpg]

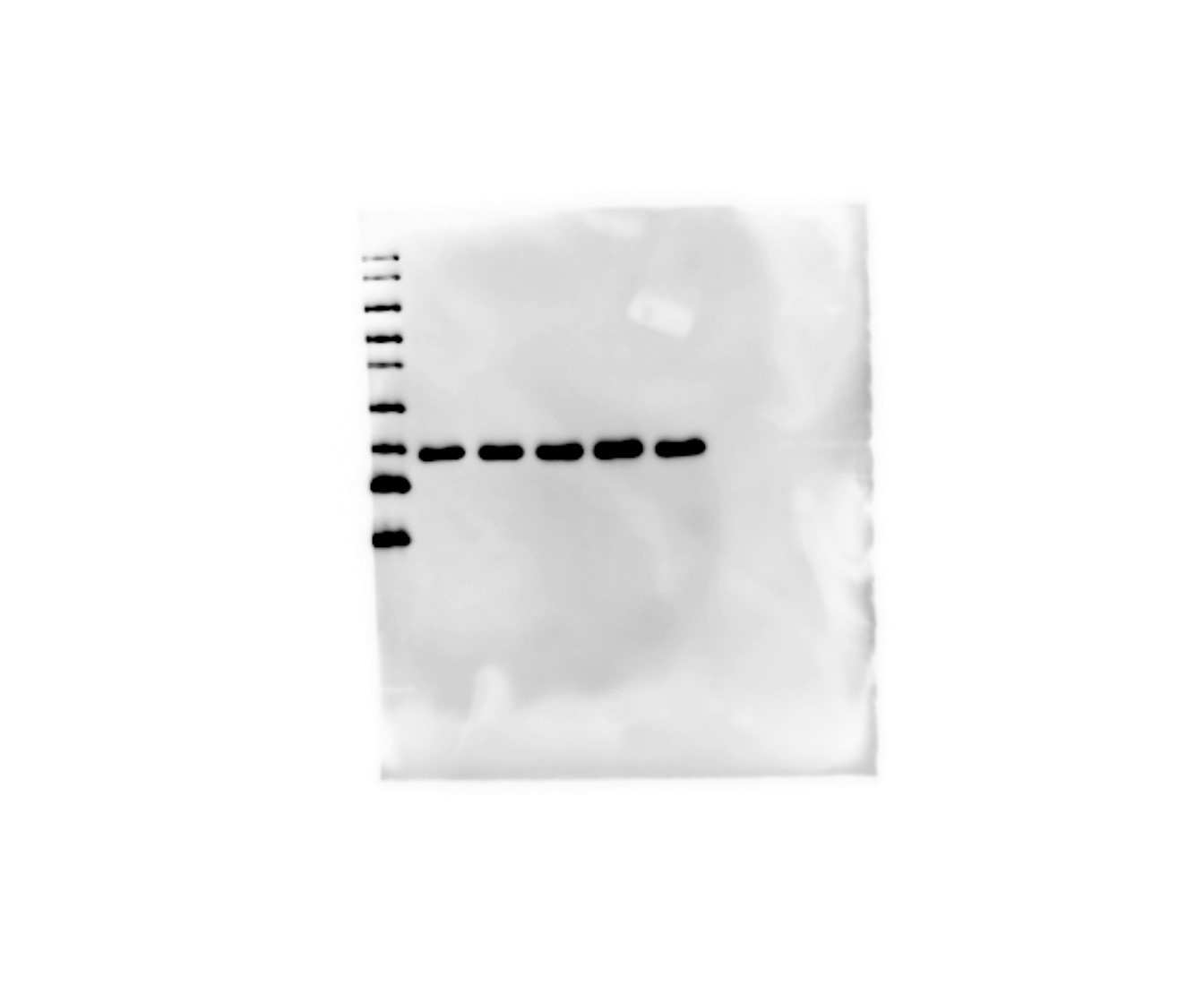

Supplement: Supplementary file 1 — Supplementary Information. [file 41598_2023_49994_MOESM1_ESM.zip › Fig4A SO-RB50 GAPDH.jpg]

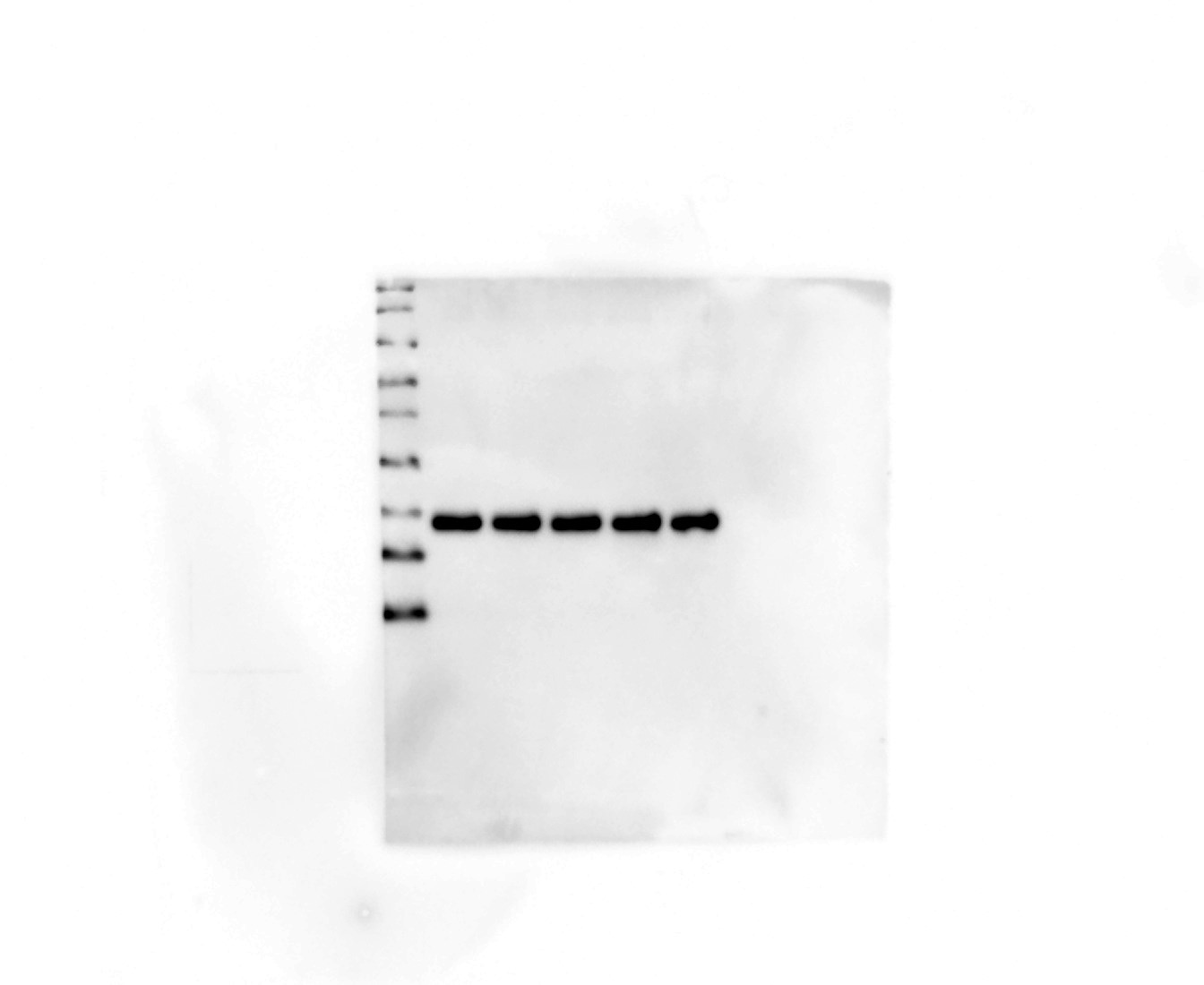

Supplement: Supplementary file 1 — Supplementary Information. [file 41598_2023_49994_MOESM1_ESM.zip › Fig4D HXO-RB44 GAPDH.jpg]

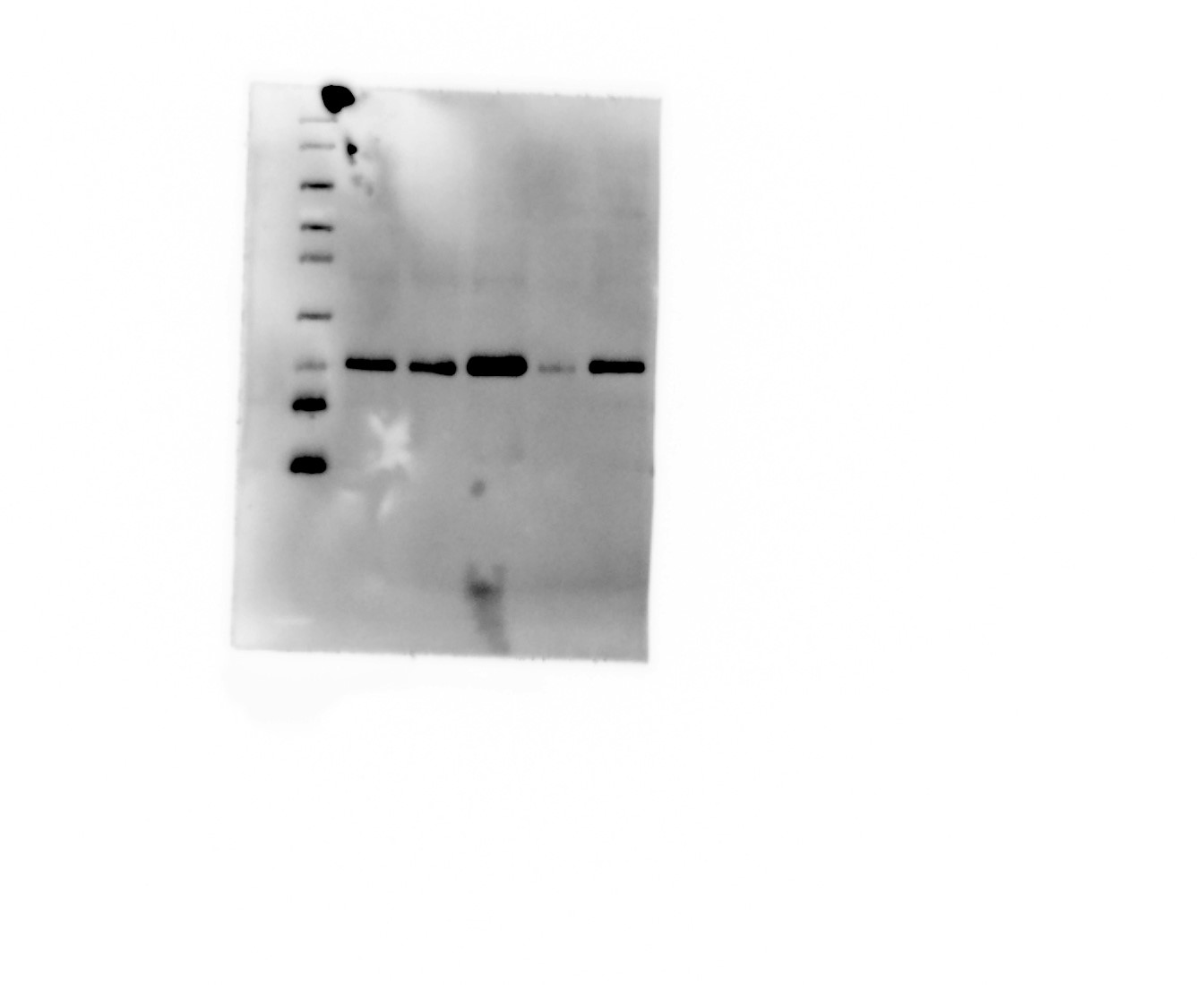

Supplement: Supplementary file 1 — Supplementary Information. [file 41598_2023_49994_MOESM1_ESM.zip › Fig4D HXO-RB44 p-p38MAPK.jpg]

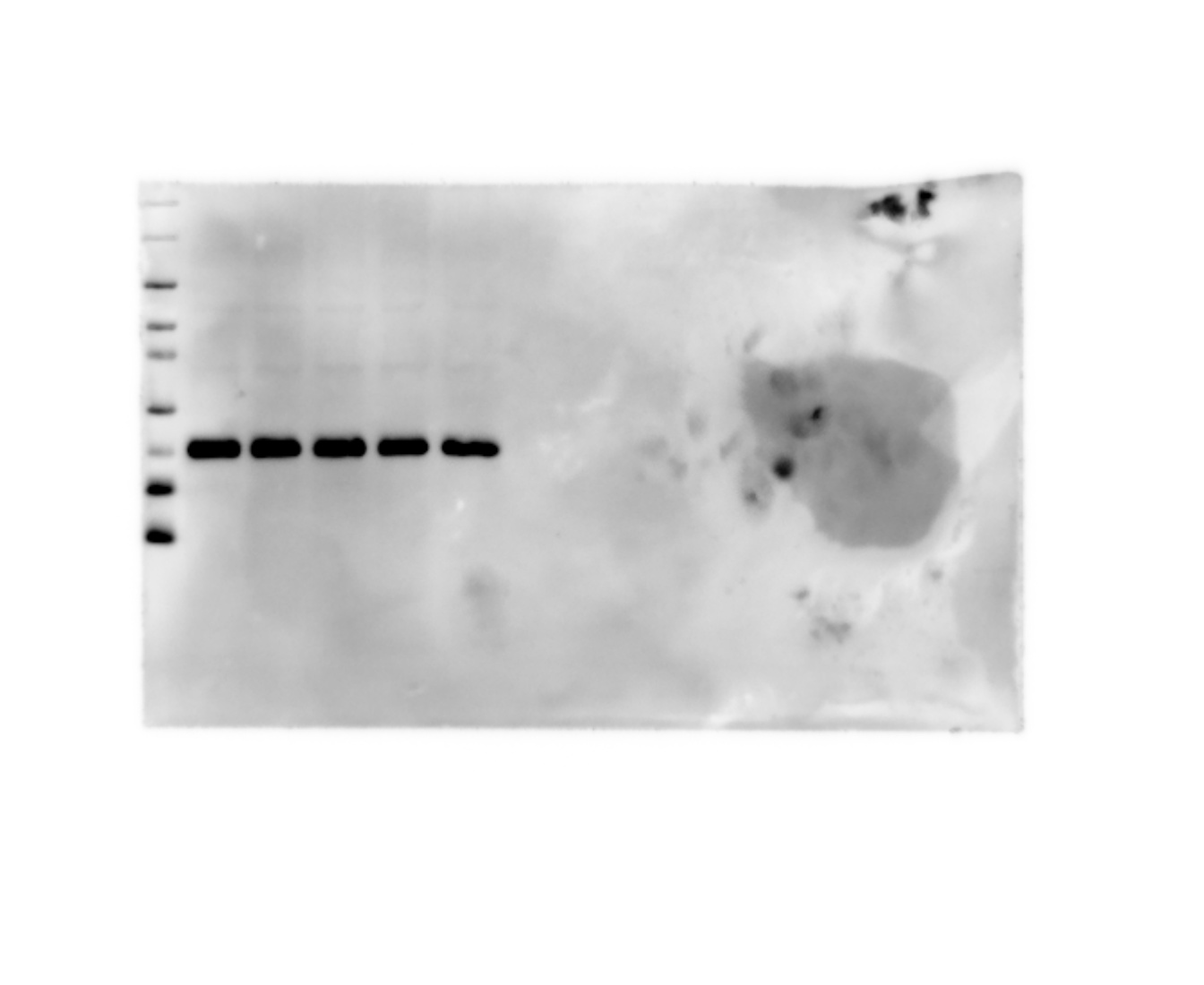

Supplement: Supplementary file 1 — Supplementary Information. [file 41598_2023_49994_MOESM1_ESM.zip › Fig4D HXO-RB44 p38MAPK.jpg]

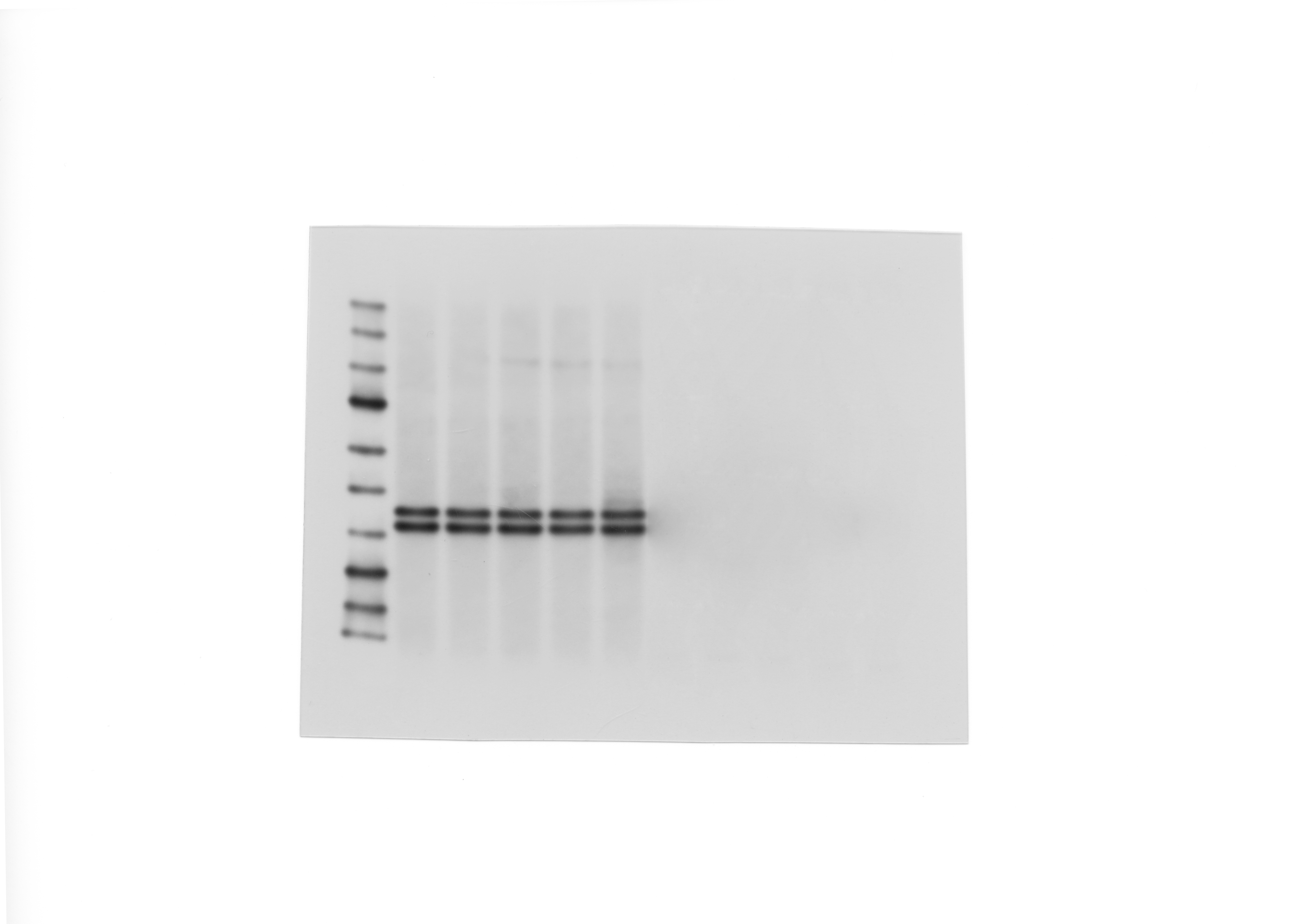

Supplement: Supplementary file 1 — Supplementary Information. [file 41598_2023_49994_MOESM1_ESM.zip › Fig4D HXO-RB44 ERK.jpg]

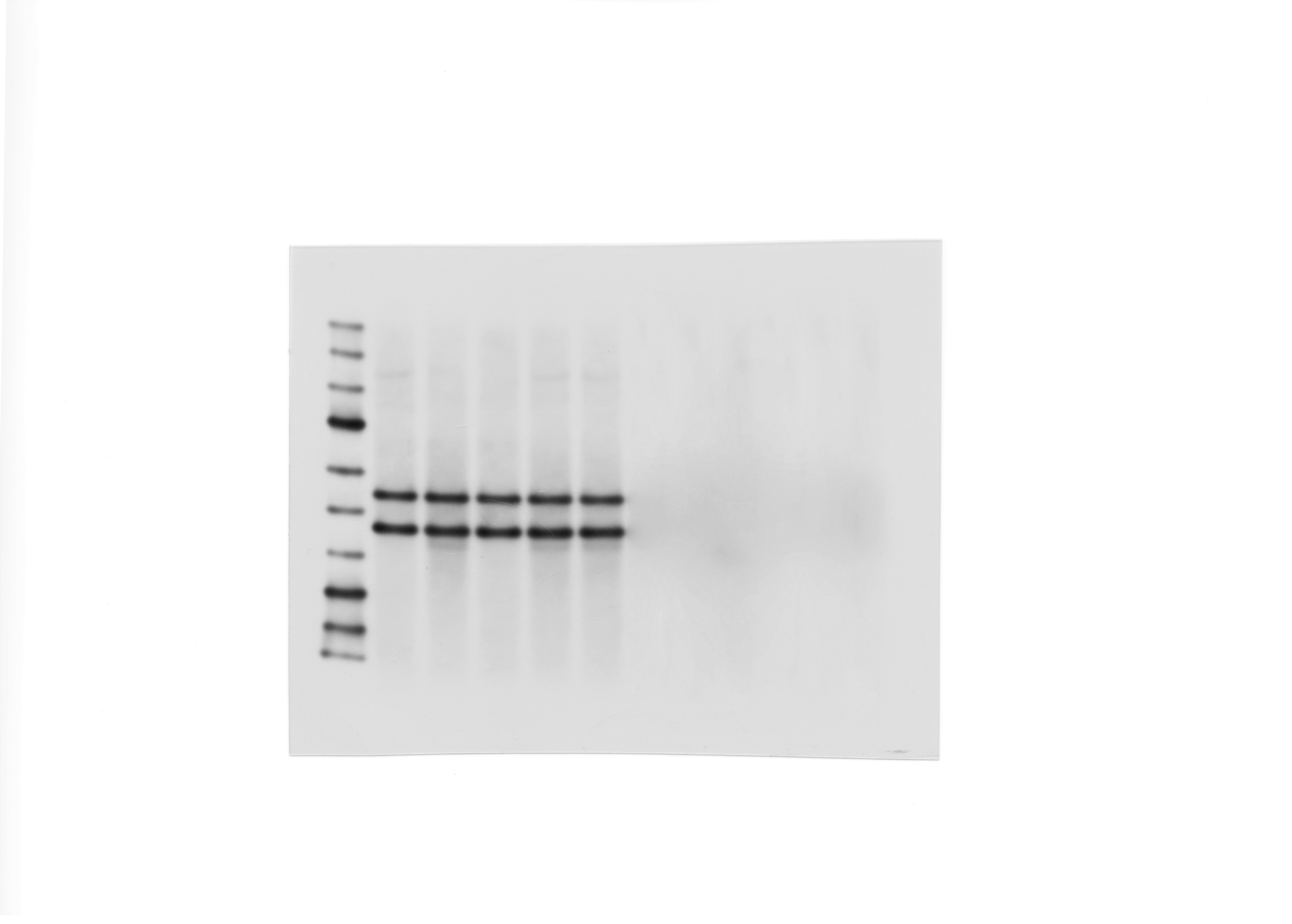

Supplement: Supplementary file 1 — Supplementary Information. [file 41598_2023_49994_MOESM1_ESM.zip › Fig4D HXO-RB44 JNK.jpg]

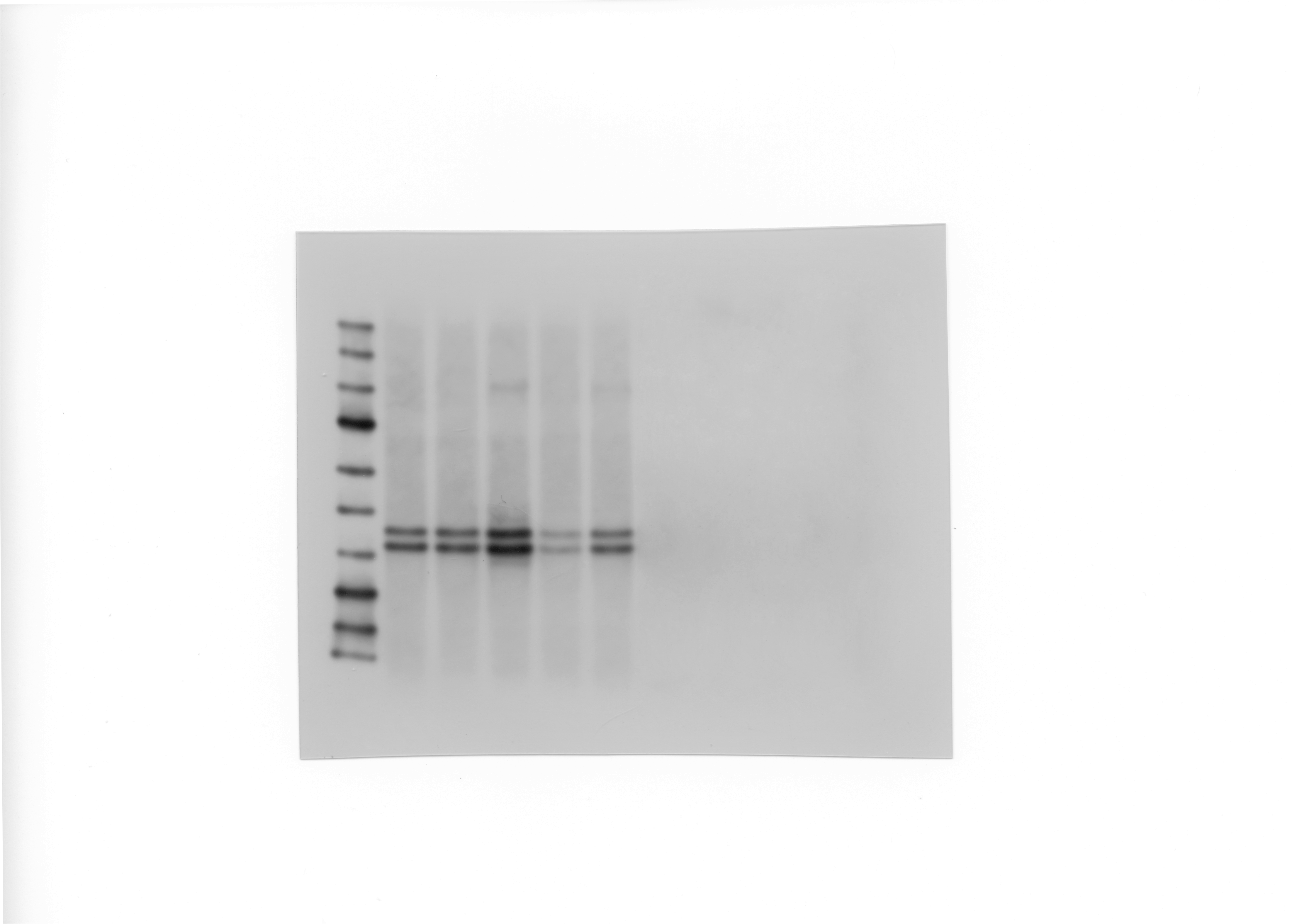

Supplement: Supplementary file 1 — Supplementary Information. [file 41598_2023_49994_MOESM1_ESM.zip › Fig4D HXO-RB44 p-ERK.jpg]

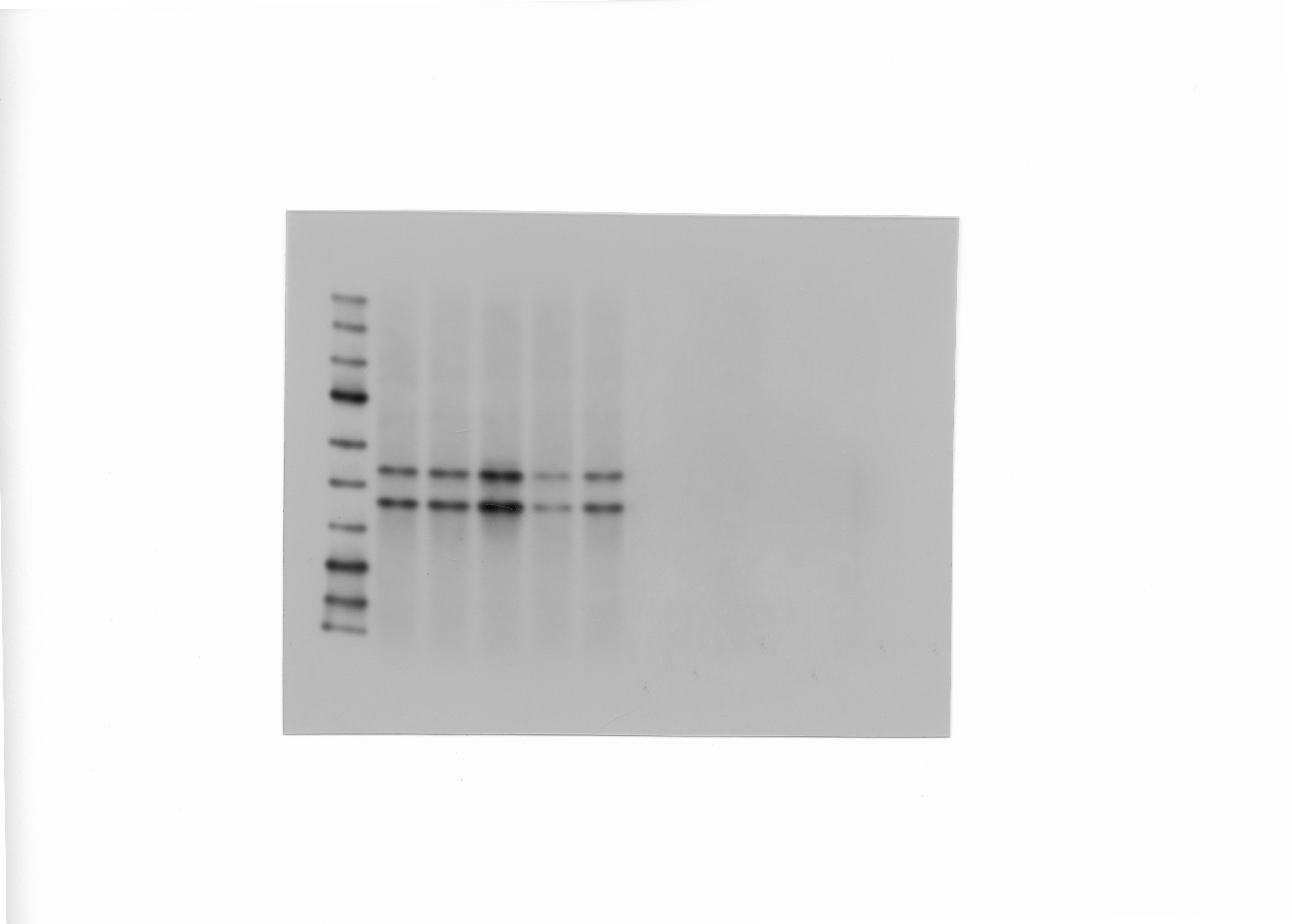

Supplement: Supplementary file 1 — Supplementary Information. [file 41598_2023_49994_MOESM1_ESM.zip › Fig4D HXO-RB44 p-JNK.jpg]

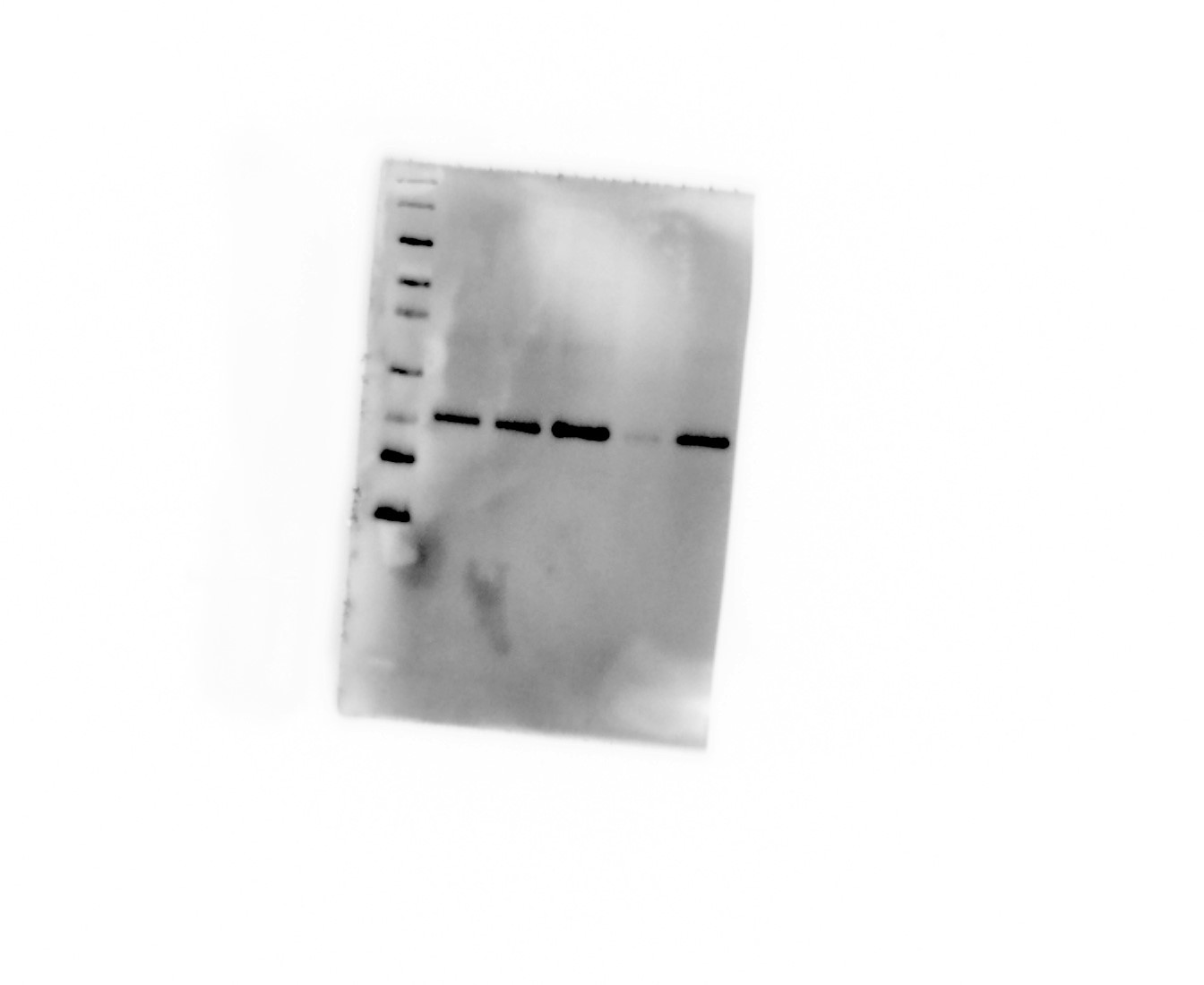

Supplement: Supplementary file 1 — Supplementary Information. [file 41598_2023_49994_MOESM1_ESM.zip › Fig4D SO-RB50 p-p38MAPK.jpg]

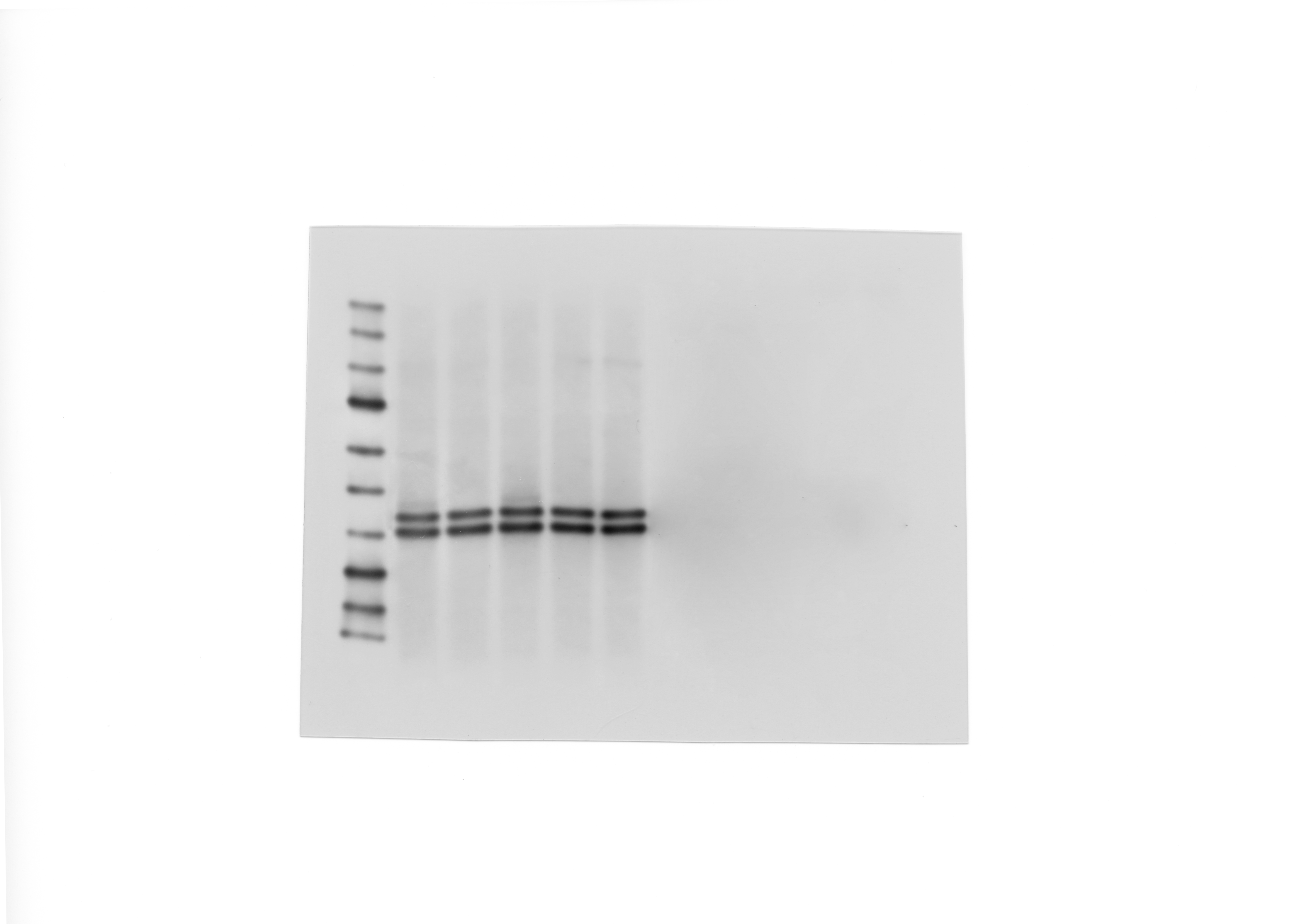

Supplement: Supplementary file 1 — Supplementary Information. [file 41598_2023_49994_MOESM1_ESM.zip › Fig4D SO-RB50 ERK.jpg]

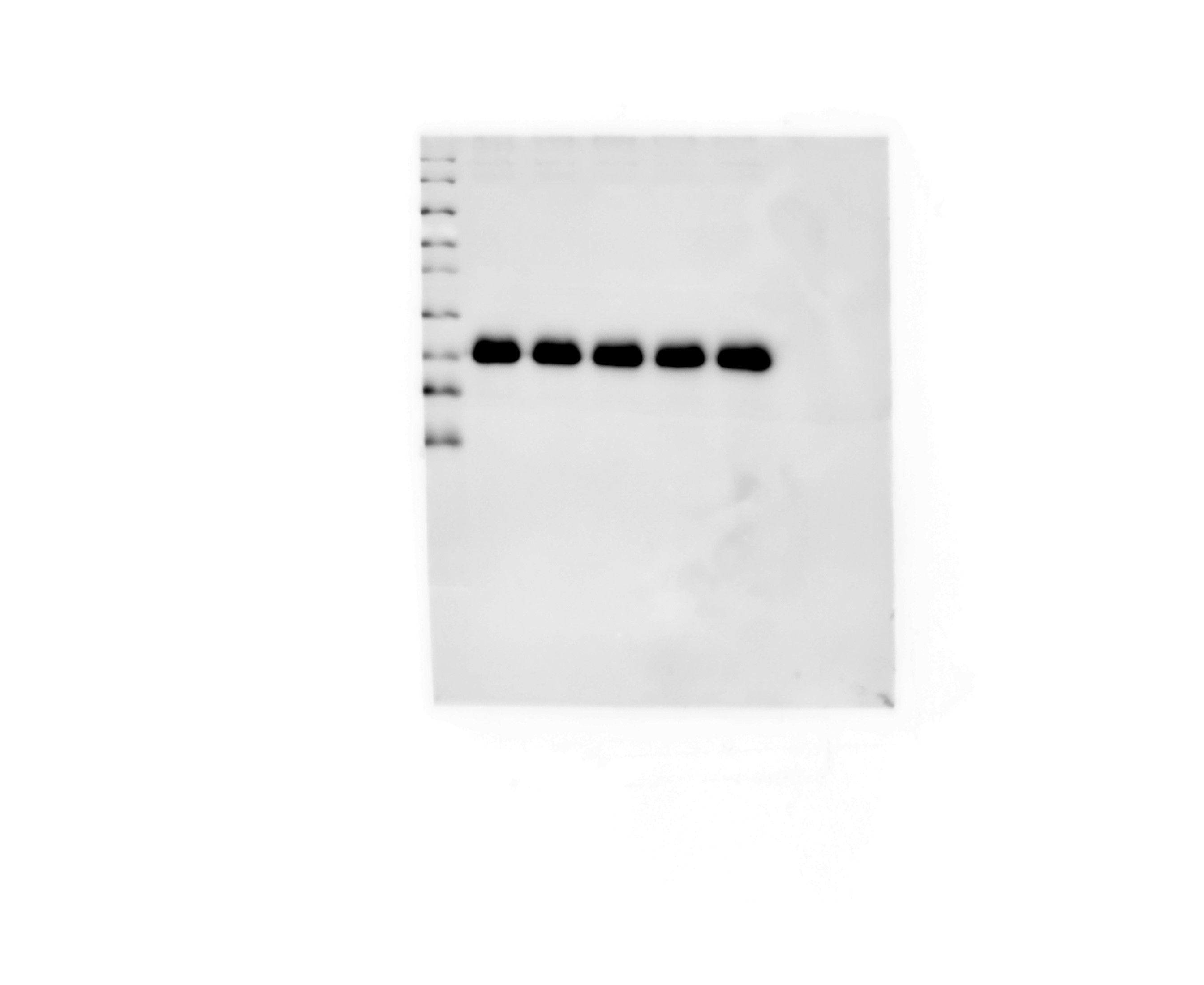

Supplement: Supplementary file 1 — Supplementary Information. [file 41598_2023_49994_MOESM1_ESM.zip › Fig4D SO-RB50 GAPDH.jpg]

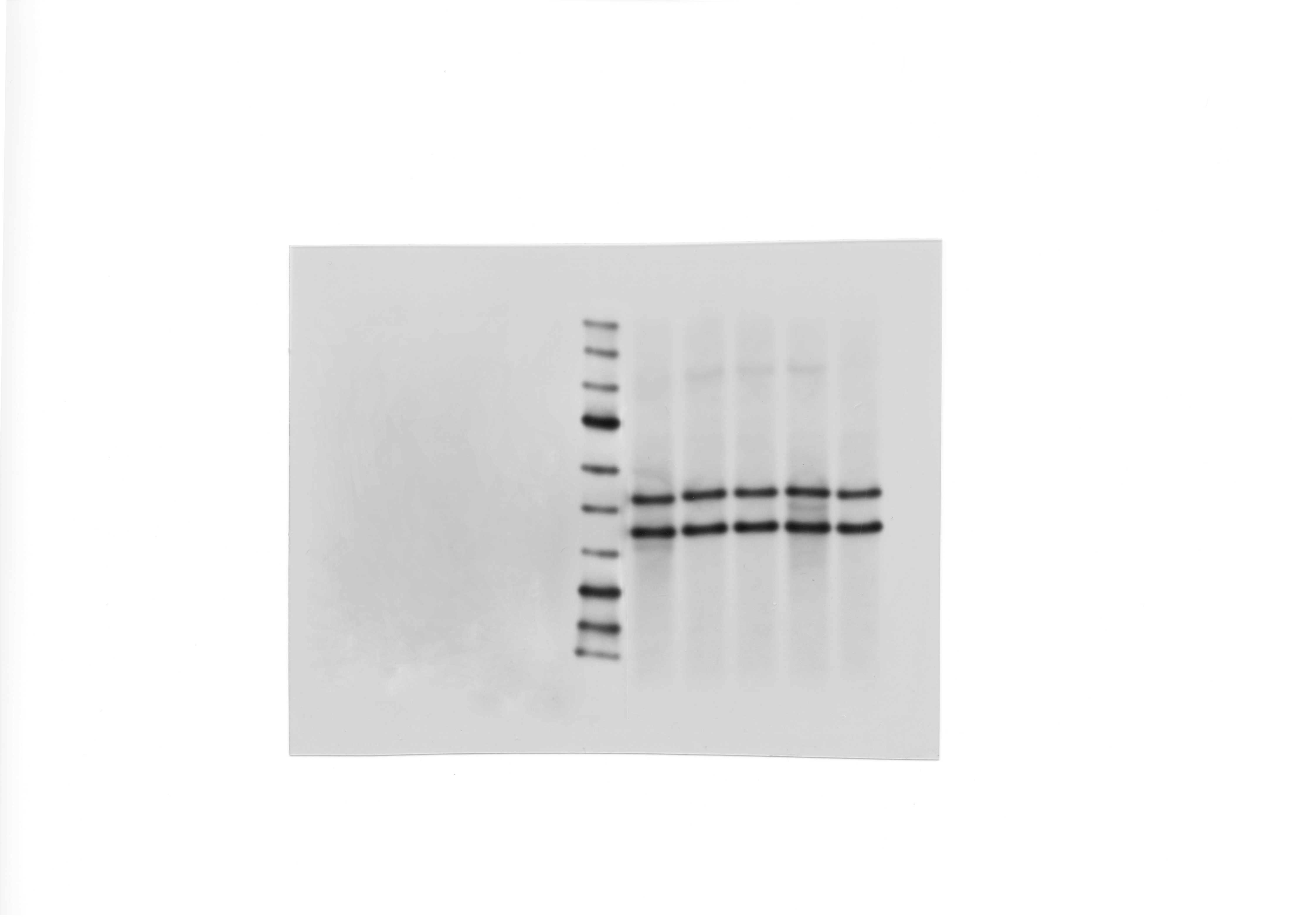

Supplement: Supplementary file 1 — Supplementary Information. [file 41598_2023_49994_MOESM1_ESM.zip › Fig4D SO-RB50 JNK.jpg]

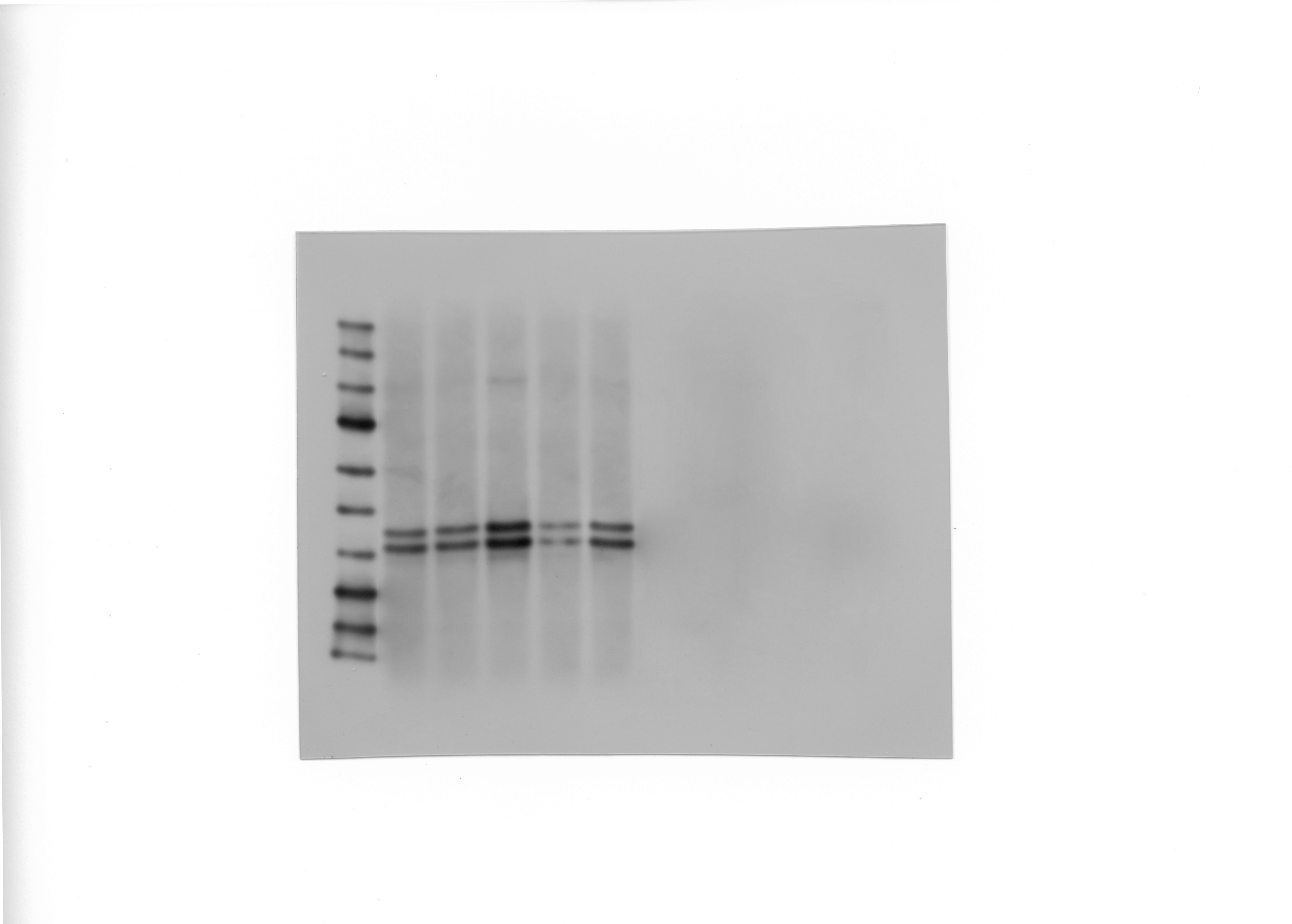

Supplement: Supplementary file 1 — Supplementary Information. [file 41598_2023_49994_MOESM1_ESM.zip › Fig4D SO-RB50 p-ERK.jpg]

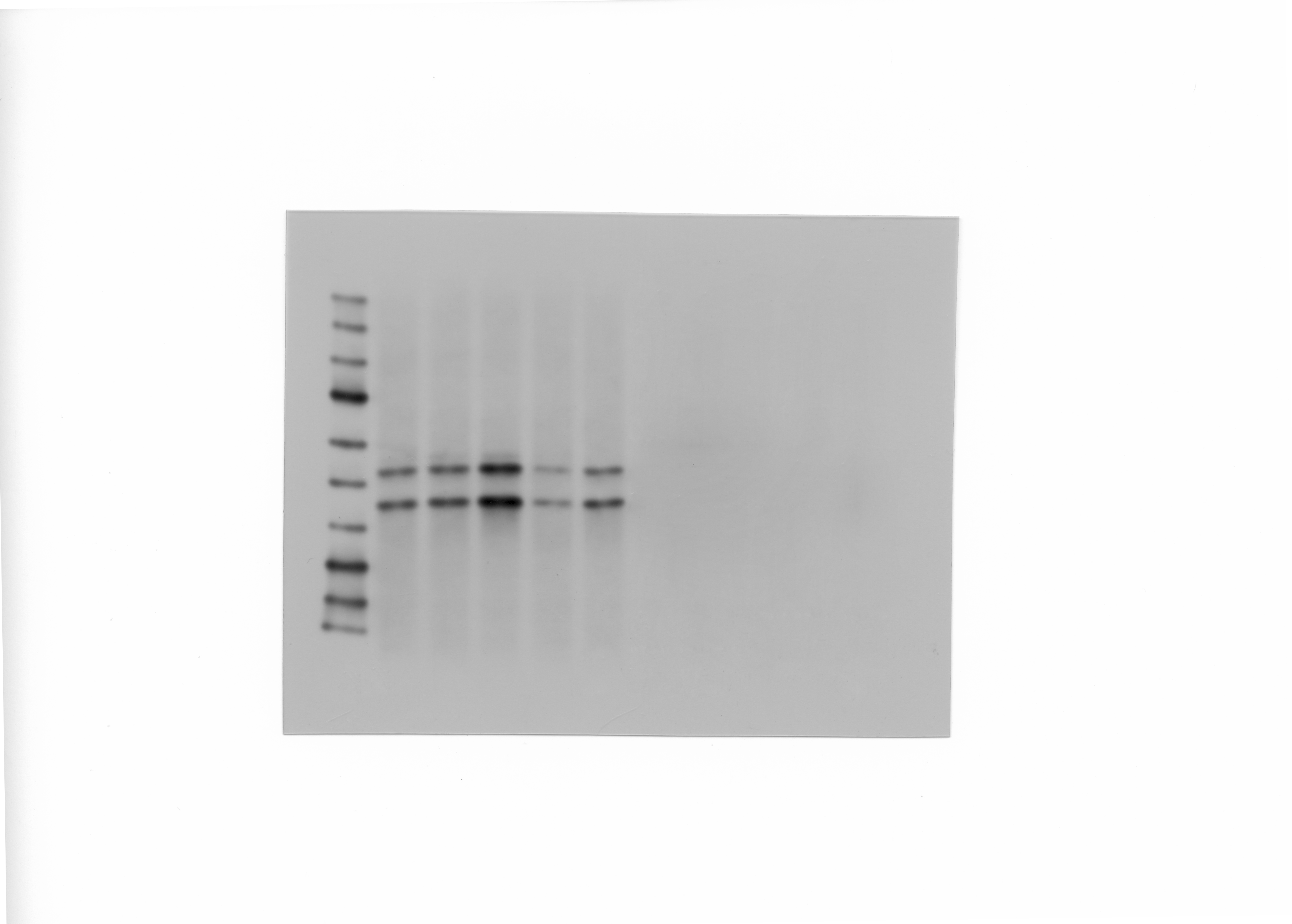

Supplement: Supplementary file 1 — Supplementary Information. [file 41598_2023_49994_MOESM1_ESM.zip › Fig4D SO-RB50 p-JNK.jpg]

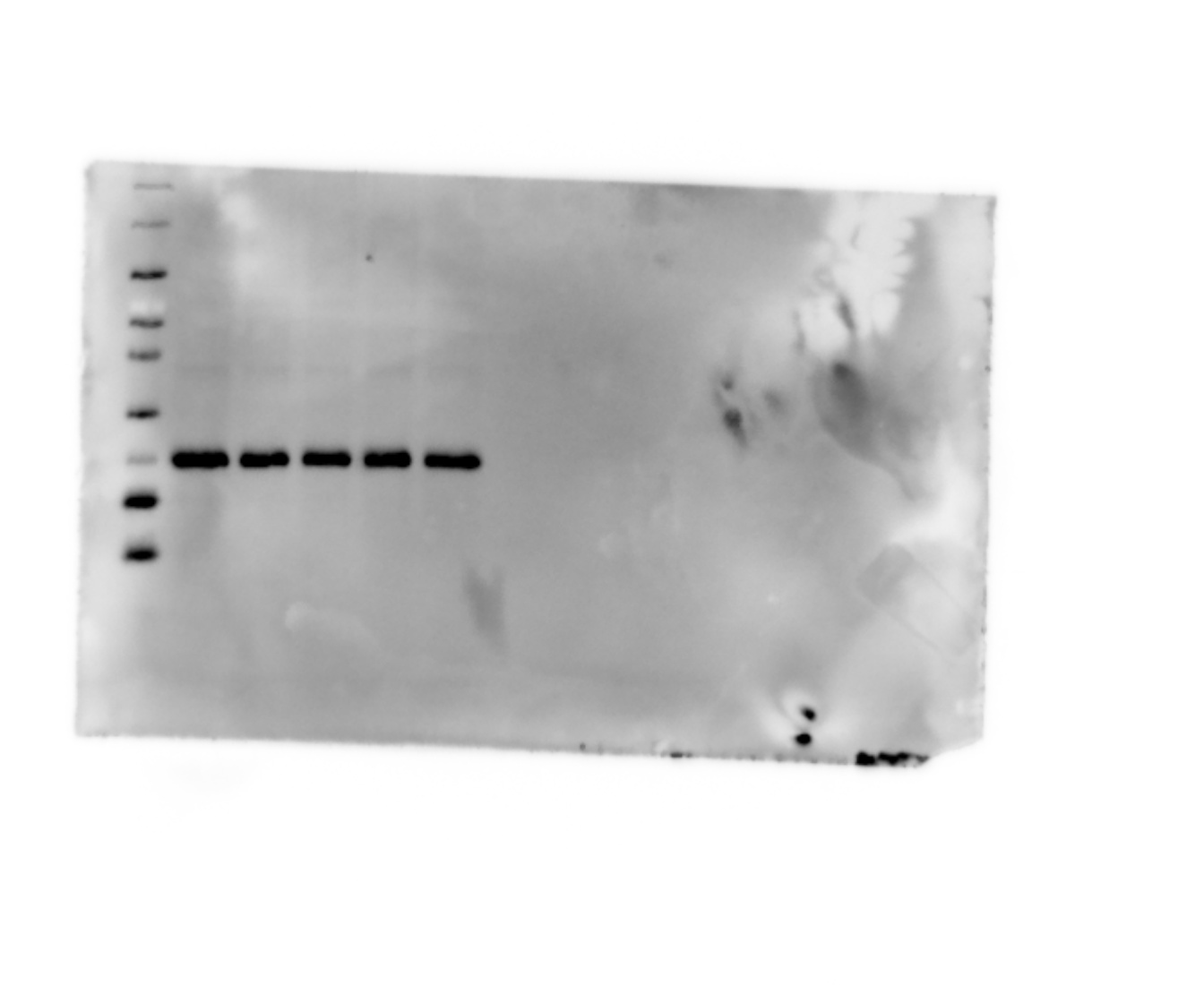

Supplement: Supplementary file 1 — Supplementary Information. [file 41598_2023_49994_MOESM1_ESM.zip › Fig4D SO-RB50 p38MAPK.jpg]
